# Analysis and Risks of Emerging Contaminants and Microplastics in Natural and Treated Waters and Human Health: A Critical Review

**DOI:** 10.3390/jox16030093

**Published:** 2026-05-23

**Authors:** Maryam Mallek, Damià Barceló

**Affiliations:** 1 Laboratory of Material Sciences and Environment, Faculty of Sciences, University of Sfax, Route de la Soukra Km 3.5, BP 1171, Sfax 3000, Tunisia; 2 Department de Química, Facultat de Ciències, Universitat de Girona, M Aurèlia Capmany, 69, 17003 Girona, Spain; 3 Chemistry and Physics Department, University of Almeria, Ctra Sacramento s/n, 04120 Almería, Spain; 4 Sino-Spain Joint Laboratory for Agricultural Environment Emerging Contaminants of Zhejiang Province, Zhejiang A&F University, Hangzhou 311300, China

**Keywords:** emerging contaminants, microplastics, high-resolution mass spectrometry, suspect screening, non-target screening, water monitoring, risk assessment

## Abstract

Emerging contaminants (ECs) and microplastics (MPs) are increasingly detected in surface waters, wastewaters, and drinking water, often as complex mixtures, transformation products, and particle-associated burdens that challenge routine monitoring. This critical review examines current analytical strategies for the detection and characterization of both molecular and particulate emerging contaminants in aquatic systems, with particular emphasis on their relevance to environmental and human health risk assessment. For molecular ECs, targeted LC–MS/MS and GC–MS and GC–MS/MS approaches are evaluated alongside high-resolution mass spectrometry (HRMS)-based suspect and non-target screening, retrospective data mining, and transformation-product elucidation. For MPs, particle-resolved vibrational spectroscopy including µ-FTIR and µ-Raman is critically assessed in comparison with complementary thermal analysis methods, such as pyrolysis–GC–MS and thermal extraction–desorption GC–MS (TED–GC–MS). Particular attention is given to the influence of sampling design, matrix-adapted sample preparation, analytical confidence, and method-dependent size and polymer coverage on data quality and interstudy comparability. The review further highlights the risks of ECs in relation to exposure pathways, mixture effects, and the potential carrier role of MPs for ECs, additives, and microorganisms. Finally, key priorities are identified for next-generation monitoring frameworks, including harmonized workflows, transparent confidence reporting, and stronger integration of analytical evidence with fate, exposure, and risk assessment.

## 1. Introduction

The occurrence of emerging contaminants (ECs) and microplastics (MPs) in aquatic environments has become a major challenge for environmental analytical chemistry thanks to their expanding diversity, widespread detection, and potential relevance for ecosystem and human health [1,2]. This challenge is further intensified by water scarcity, wastewater reclamation, and water reuse practices, which increase opportunities for contaminant recirculation across water, soil, crops, food, and biota [3].

In this review, ECs are considered as a broad working category of chemical and biological stressors that are increasingly detected in the environment, are often not fully regulated, and require further assessment because of their occurrence, persistence, mobility, transformation potential, or toxicity. This definition does not imply that all ECs are newly produced chemicals or that they exclude overlap with legacy persistent organic pollutants (POPs); rather, the distinction is based on current monitoring, regulatory, and risk-assessment relevance. Accordingly, the classification used here is organized according to contaminant origin, use pattern, behavior, and analytical/risk-assessment relevance, and includes pharmaceuticals and personal care products, per- and polyfluoroalkyl substances (PFAS), disinfection by-products (DBPs), endocrine-disrupting chemicals, pesticides, algal toxins, nanomaterials, tire-derived chemicals, and antibiotic resistance genes (ARGs) [4,5,6,7,8,9,10,11,12,13]. PFAS are included as an important EC class because their strong C–F bonds contribute to their extreme persistence and resistance to environmental and treatment-induced degradation [14]. In parallel, MPs have emerged as ubiquitous particulate contaminants in surface waters, wastewaters, and drinking water systems, raising concern not only because of their physical presence, but also because of their interactions with additives, sorbed pollutants, metals, and microorganisms [15]. Their environmental relevance is further underscored by large-scale plastic pollution inputs to aquatic environments, particularly from fragmented and single-use plastics [16].

Conventional wastewater and drinking water treatment processes were not originally designed to remove many of these contaminants, which contributes to their continued release into receiving waters and, in some cases, their entry into drinking water supplies [11,12]. As a result, ECs and MPs are frequently detected at trace and ultra-trace levels, often as complex mixtures that complicate both analytical determination and risk assessment [15,16]. In urban receiving waters, this challenge is amplified by short-lived episodic inputs, such as storm-driven pulses of 6PPD-quinone (6PPDQ), which highlight the limitations of single grab samples for capturing peak exposure conditions [17]. For MPs, environmental concern is further reinforced by the increasing contribution of land-based sources, including sewage sludge reuse, wastewater irrigation, biosolids, composting, and fragmentation of mulching films and other plastic wastes, to MP inputs in agroecosystems [18]. These concerns are further complicated by the formation of transformation products during treatment and environmental processes, particularly for pharmaceuticals, pesticides, PFAS precursors, and DBPs, some of which may persist, evade routine monitoring, and exhibit toxicity comparable to or greater than that of their parent compounds [11,19,20,21].

From an analytical perspective, molecular and particulate emerging contaminants present distinct but complementary challenges because they are measured according to different analytical principles. Molecular ECs are dissolved or extractable chemical species, and their assessment depends mainly on selective extraction, chromatographic separation, ionization efficiency, mass-spectrometric detection, matrix-effect correction, and confirmation using retention time, precursor/product ions, accurate mass, or MS/MS spectra [22]. Targeted analysis based on liquid chromatography–tandem mass spectrometry (LC–MS/MS) and gas chromatography–tandem mass spectrometry (GC–MS/MS) remains the reference approach for the quantitative determination of predefined contaminants; however, these methods are restricted to known analytes and cannot fully address the continuously evolving chemical space of emerging pollutants [23,24]. In contrast, high-resolution mass spectrometry (HRMS) has expanded analytical capabilities through suspect and non-target screening, enabling the detection, structural annotation, and retrospective interrogation of known, unknown, and newly recognized contaminants, including transformation products, novel PFAS, and rubber-derived chemicals [25,26,27,28]. Key pain points for molecular EC analysis include matrix suppression/enhancement, lack of authentic standards, incomplete target lists, uncertainty in non-target identification, and limited comparability among studies using different workflows.

MPs, however, are particulate emerging contaminants whose analysis relies on fundamentally different principles. Their characterization requires physical isolation and particle-resolved or mass-based determination of particle number, size, shape, color, morphology, and polymer composition. Spectroscopic techniques such as µ-FTIR and Raman microscopy provide particle-resolved polymer identification and morphology information, whereas thermal methods such as pyrolysis–GC–MS and thermal extraction–desorption GC–MS provide polymer-mass information but generally do not preserve particle number, size, or shape [29]. Therefore, the main analytical challenges for MPs are not ionization or chromatographic selectivity, but representative sampling, contamination control, matrix digestion, density separation, particle-size cut-off, polymer confirmation, recovery validation, and harmonized reporting units. Current workflows remain method-dependent and incomplete, as no single technique can yet provide comprehensive characterization across all environmentally relevant particle-size classes and matrix types. Interlaboratory studies have further highlighted harmonization as a major unmet need, particularly for small MPs and nanoplastics [30,31]. This contrast between chemical species analysis for molecular ECs and particle-resolved characterization for MPs explains why integrated monitoring requires complementary workflows rather than a single analytical platform. In addition, apparent “removal” from water often reflects transfer to solids rather than true elimination, underscoring the need for comparable analytical strategies across both aqueous and solid compartments [32].

Beyond analytical detection, increasing attention is being paid to the environmental and human health implications of ECs and MPs. Chronic exposure to low concentrations of EC mixtures, including endocrine disruptors, PFAS, pesticides, and pharmaceutical residues, raises concerns about endocrine disruption, antimicrobial resistance, and long-term toxicity [5,6,10,33]. For MPs, potential risks arise from their physical presence, associated chemical additives, and their potential role as carriers of organic contaminants, metals, and microorganisms, including resistance determinants [34,35,36]. Under water reuse and food production scenarios, these concerns may extend beyond aquatic exposure alone to include transfer through soil–plant systems, trophic pathways, and ultimately human dietary exposure [34,37,38]. Nevertheless, risk assessment remains strongly constrained by analytical uncertainties, incomplete chemical characterization, and limited toxicological evidence, underscoring the need for more integrated analytical-toxicological frameworks.

**Figure 1:**
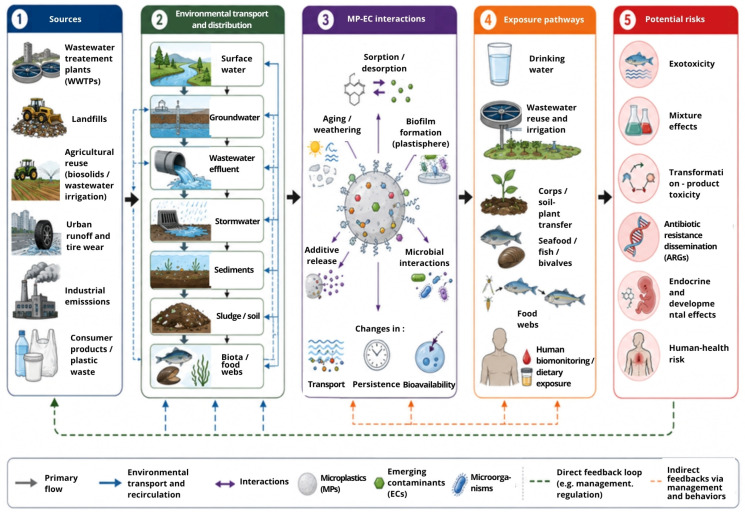
Conceptual overview of EC and MP pathways, interactions, exposure, and risks. Major sources include wastewater treatment plants, landfills, agricultural reuse, urban runoff and tire wear, industrial emissions, and consumer products/plastic wastes. ECs and MPs are transported through surface water, groundwater, wastewater effluent, stormwater, sediments, sludge/soil, and biota/food webs. MPs may interact with ECs and microorganisms through sorption/desorption, aging/weathering, biofilm formation, additive release, and microbial interactions, thereby modifying contaminant transport, persistence, and bioavailability. These processes contribute to exposure through drinking water, wastewater reuse, crops, seafood, food webs, and biomonitoring, with potential outcomes including ecotoxicity, mixture effects, transformation-product toxicity, antibiotic resistance dissemination, endocrine and developmental effects, and human health risks.

In this context, the present review critically evaluates current analytical strategies for the determination of molecular and particulate ECs in natural and treated waters. Particular emphasis is placed on how recent analytical advances, especially HRMS-based suspect and non-target screening strategies and complementary particle-resolved approaches for MPs, can improve the reliability of occurrence assessment and strengthen the integration of monitoring data with environmental and human health risk evaluation. Sampling and sample preparation strategies, targeted and non-target analytical methods, and emerging directions such as retrospective data mining and transformation-product analysis are examined. By identifying current limitations, methodological gaps, and future analytical priorities, this review aims to support the development of harmonized, robust, and risk-relevant frameworks for the monitoring and assessment of ECs and MPs in aquatic systems. Figure 1 provides a conceptual overview of the relationships among EC and MP sources, environmental transport pathways, MP–EC interactions, exposure routes, and potential environmental and human health risks, thereby framing the integrated analytical and risk-assessment perspective developed throughout this review.

Literature search strategy. The literature included in this review was selected through a critical search of major journals and databases in environmental science, analytical chemistry, toxicology, and water research, including Web of Science, Scopus, ScienceDirect, ACS Publications, PubMed, SpringerLink, Wiley Online Library, MDPI, and Google Scholar. The selection was also guided by the authors’ previous expertise in emerging contaminants, microplastics, environmental analysis, and toxicological risk assessment. Most references correspond to peer-reviewed studies published during the last five years, although earlier papers were retained when they provided essential methodological or conceptual background. The main keywords included “emerging contaminants”, “contaminants of emerging concern”, “microplastics”, “nanoplastics”, “water monitoring”, “wastewater”, “LC–MS/MS”, “GC–MS/MS”, “HRMS”, “suspect screening”, “non-target screening”, “µ-FTIR”, “µ-Raman”, “pyrolysis–GC–MS”, “thermal extraction–desorption GC–MS”, “risk assessment”, “mixture effects”, and “human exposure”. Studies were prioritized when they provided relevant information on occurrence, sampling, sample preparation, analytical performance, environmental fate, exposure pathways, and risk assessment in natural and treated waters.

## 2. Occurrence and Sources of Emerging Contaminants and Microplastics in Water Systems

### 2.1. Molecular Emerging Contaminants

Molecular emerging contaminants occur in water systems through interconnected source pathways that include landfills, WWTPs, agricultural reuse practices, urban runoff, consumer products, industrial emissions, and long-range atmospheric transport. Their occurrence is shaped not only by input intensity, but also by hydrology, partitioning behavior, transformation, and incomplete removal during treatment. As a result, aquatic environments receive complex mixtures of antibiotics and other pharmaceuticals and personal care products (PPCPs), PFAS, tire-derived chemicals, cyanotoxins, nanomaterials, and resistance-related contaminants, often at trace levels but at higher concentrations in concentrated matrices such as landfill leachates [37,39].

Landfills are increasingly recognized as important reservoirs of both biological and chemical emerging contaminants. Metagenomic analysis of municipal solid-waste landfill refuse has revealed diverse ARG profiles associated with mobile genetic elements and heavy metals, indicating the potential for co-selection processes [40]. This biological role is paralleled by a complex chemical burden, since landfill leachates may contain numerous PPCPs and other contaminants, often at concentrations higher than those reported in municipal wastewater [41,42]. Their composition is also hydrologically variable, with contaminant loads changing between wet and dry periods [43].

Compared with WWTP effluents, landfills therefore represent less controlled but often more concentrated reservoirs capable of releasing both resistance determinants and high contaminant loads to surrounding waters.

WWTPs, however, are continuous but incomplete barriers, acting simultaneously as point sources and redistribution nodes. They receive sustained inputs of antibiotics and other PPCPs from domestic use, hospitals, and industry, and conventional treatment does not fully remove many of these compounds [44]. At the treatment scale, pharmaceutical mixtures may contribute to biological effects even when individual compounds occur at low concentrations, highlighting the importance of mixture-based interpretation [33]. In parallel, many PPCPs partition into primary and waste activated sludge, particularly hydrophobic and ionizable compounds such as triclosan, triclocarban, and fluoroquinolone antibiotics [45,46,47]. Therefore, apparent removal from the aqueous phase does not necessarily indicate elimination, but may reflect transfer to sludge. During sludge treatment and reuse, these retained contaminants may affect anaerobic digestion performance and create secondary release pathways through land application, digestate reuse, and runoff to soils and waters [48,49]. Thus, compared with landfill leachates, sludge represents a more concentrated but less mobile reservoir, although its agricultural use can redistribute contaminants into terrestrial and aquatic compartments.

Agricultural reuse practices extend this contamination into the soil–water–plant continuum. Treated wastewater, biosolids, digestates, and plastic-based agricultural materials can introduce PPCPs, antibiotics, PFAS, MPs, ARGs, and other contaminants into agricultural soils, where they may redistribute among bulk soil, porewater, rhizosphere compartments, and plant tissues [36,37,38,50]. Field and greenhouse studies have documented the uptake of more than 100 PPCPs under reuse scenarios and collectively show that porewater can be a better predictor of plant uptake than bulk soil concentration, while contaminant distribution often follows compound- and crop-specific patterns, with stronger retention in roots than in edible tissues for many PPCPs [38,50]. Therefore, reuse practices should be assessed across connected water, soil, porewater, crop, and biosolid compartments rather than through irrigation-water quality alone. Although concentrations in agro-food systems are generally lower than those in landfill leachates, they are more directly relevant to chronic dietary exposure and food-chain transfer [37,51].

Beyond wastewater, landfills, and agricultural reuse, additional diffuse, consumer-related, industrial, and climate-mediated pathways contribute to the occurrence of molecular ECs across aquatic systems. Within the broader PPCP group, personal care products represent a persistent diffuse source because many UV filters, preservatives, fragrances, and synthetic musks are used continuously in consumer products and can enter wastewater and solid-waste streams. Several of these compounds are hydrophobic and resistant to complete removal, favoring partitioning into sludge, sediments, and biota. Artificial sweeteners provide a complementary example of highly persistent wastewater-linked markers. Together, these consumer-derived compounds illustrate how everyday-use chemicals can contribute to sustained dissolved and particle-associated contaminant burdens in aquatic systems [52,53].

These source-driven inputs are complemented by long-range transport and climate-related remobilization, which can introduce persistent contaminants into remote and high-altitude environments. Beyond direct emission sources, remote and high-altitude ecosystems are increasingly recognized as reservoirs of persistent and emerging contaminants. High-mountain Alpine lakes act as “cold traps” for POPs and selected contaminants of emerging concern, including PCBs, DDTs, PBDEs, PAHs, synthetic musks, PFAS and MPs. Despite minimal local anthropogenic activity, atmospheric transport, glacial meltwater remobilization, and seasonal deposition promote accumulation in sediments and biota. Among the reviewed studies, sediments were the dominant matrix (≈65%), followed by fish (≈33%), whereas water was rarely investigated (≈2%), which is consistent with the strong partitioning of hydrophobic contaminants [54]. Sediment-core records further revealed secondary concentration increases associated with glacier retreat, indicating cryospheric re-release of stored pollutants. In the same context, aerosol-bound C_60_ and C_70_ were detected in 43 Mediterranean marine-air samples, with median concentrations of 0.06 and 0.48 ng m^−3^, respectively, and a maximum C_70_ concentration of 233.8 ng m^−3^, showing that engineered nanomaterials, like legacy contaminants, can also undergo long-range atmospheric dispersal [55].

Long-range transport and climate forcing are likewise reflected in biological and bloom-mediated exposure pathways. In polar bears from Western and Southern Hudson Bay, 109 PCA homologues spanning C8–C26 were detected, with ΣPCA concentrations up to 260 ng g^−1^ lipid. However, temporal trends diverged between subpopulations, suggesting that contaminant burdens are modulated not only by atmospheric transport but also by climate-driven dietary shifts [56]. Cyanotoxins represent a different but equally climate-sensitive occurrence pattern: under eutrophic and thermally stable conditions, microcystin concentrations frequently exceed the WHO provisional drinking-water guideline of 1 µg L^−1^, and multiple toxin families, including microcystins (MCs), cylindrospermopsin (CYN), anatoxin-a (ATX), and nodularin (NOD), often co-occur in bloom-impacted waters [56,57]. Thus, compared with remote contaminant accumulation driven by deposition and remobilization, cyanotoxin occurrence reflects in situ production enhanced by changing environmental conditions.

In urban environments, by contrast, traffic-related sources generate more episodic and pulse-like contamination patterns, especially during rainfall and stormwater runoff events. Urban runoff is a major pathway for tire-derived contaminants, including 6PPD-quinone (6PPDQ), benzothiazoles, and related rubber additives. In multi-site monitoring, 6PPDQ ranged from ~1.3 ng L^−1^ to 75 µg L^−1^ in roadway runoff, illustrating the broad concentration variability associated with storm-driven mobilization [58,59,60]. These compounds are released during tire wear, transformed during environmental exposure, and transported as mixtures of parent additives, oxidation products, metals, and transformation products [61,62,63,64].

Beyond stormwater runoff, wastewater effluents, rubber manufacturing emissions, recycled rubber materials, and legacy tire deposits can provide more continuous or long-term sources of tire-derived chemicals [24,61,65]. This distinction is important because episodic runoff mainly drives short-term exposure pulses, whereas industrial, wastewater, and legacy sources may sustain lower but more persistent background contamination. Overall, tire-derived ECs illustrate the need for event-based monitoring, non-target screening of emerging transformation products, and toxicological prioritization, especially because the reported acute LC_50_ of 6PPDQ for coho salmon is 95 ng L^−1^ and may overlap with environmentally relevant stormwater concentrations [66,67,68,69,70].

Legacy disposal of whole tires also represents an overlooked but persistent source of tire-derived chemicals. The investigation of an underwater tire dump in Hjelmås Bay (Norway), where tires had remained submerged for more than 50 years, revealed elevated sediment concentrations of Zn, Pb, and Cu near the dump center, with Zn as the dominant metal [12]. Bis(2-ethylhexyl) phthalate (DEHP) was the predominant phthalate in sediments, while non-target LC–HRMS screening of overlying waters identified 20 features potentially linked to tire degradation, including N,N′-diphenylguanidine (DPG). Unlike storm-driven urban pulses, these results indicate that submerged whole tires can act as long-term marine reservoirs of trace metals and additive-derived compounds.

Engineered nanomaterials require separate consideration because their environmental behavior depends on particle number, size, aggregation, and transformation rather than dissolved concentration alone. Single-particle ICP–MS studies have shown that Ti-, Ce-, and Ag-bearing nanoparticles occur in river basins and adjacent coastal waters, with Ag-NPs more clearly associated with WWTP-impacted hotspots, whereas Ti- and Ce-bearing particles may also reflect natural mineral backgrounds [39]. During wastewater treatment, nanoparticles can undergo substantial speciation changes; for example, AgNPs are frequently transformed into Ag_2_S, while ZnO nanoparticles may convert to ZnS, Zn_3_(PO_4_)_2_, or Fe-associated phases [71,72]. Such transformations can reduce dissolved metal release but do not necessarily eliminate persistence, bioavailability, or trophic transfer, as field and mesocosm studies have shown continued accumulation in sediments, plants, fish, and invertebrates [73,74]. Consumer products, including textiles, toothpastes, sunscreens, and food-grade TiO_2_, further contribute to environmental TiO_2_ inputs, which may differ structurally from model nanoparticles used in laboratory fate studies [75,76,77]. Overall, engineered nanomaterials illustrate the need for particle-resolved monitoring capable of distinguishing anthropogenic nanoparticles from natural colloids and tracking transformation during treatment and environmental aging.

PFAS represent a particularly important class of persistent and mobile ECs, with occurrence patterns shaped by industrial production, municipal wastewater inputs, precursor transformation, replacement chemistry, and contaminated soils. Industrial production is a major source, with PTFE-associated nonpolymeric PFAS emissions in China estimated at 1.06 × 10^5^ kg in 2023, mostly originating from the production stage [78]. Municipal WWTPs also remain persistent PFAS sources, with increases in some effluent PFAS attributed to precursor transformation during treatment [79]. Additional evidence from e-waste recycling soils shows that PFAS contamination can extend to solid matrices and may be dominated by ultrashort-chain species, particularly trifluoroacetic acid [80].

The ongoing shift from legacy to replacement PFAS is also evident in biomonitoring and hotspot studies. In U.S. serum collected between 2003 and 2021, legacy PFAS declined markedly, whereas suspect screening revealed increasing replacement compounds such as F-53B, 6:2 DiPAP, and previously unreported chloroperfluorononylphosphonic acid [81]. In southern Lyon, a fluoropolymer/fluorotelomer industrial hotspot, 22 of 47 water samples exceeded 100 ng L^−1^ Σ77PFAS, maxima reached ~700 ng L^−1^, and 67% of tap-water samples exceeded the EU 100 ng L^−1^ benchmark for Σ20PFAS; profiles were dominated by short-chain C4–C8 PFCAs, with the highest mean Σ77PFAS concentrations in groundwater (147 ng L^−1^) [82]. Together, these findings confirm that PFAS occurrence is increasingly shaped by industrial source signatures, precursor transformation, and expanding replacement chemistry.

A comprehensive monitoring study in the Guadiaro River basin (southern Spain) illustrates how molecular EC occurrence can remain substantial even in moderately anthropized watersheds overlapping protected areas. Among 171 target organic contaminants analyzed in groundwater and surface waters, 25 were detected at least once, including pharmaceuticals, drugs of abuse, and PAHs. Cocaine and its main metabolite occurred in 85% and 95% of samples, respectively, at 0.001–0.18 and 0.004–0.6 μg L^−1^, whereas pyrene was detected in all samples at 0.001–0.015 μg L^−1^ [83]. Notably, concentrations and calculated risk quotients were generally higher in groundwater, especially in detrital aquifers, indicating greater accumulation and slower hydrodynamics than in karst systems. This contrasts with the more episodic patterns often observed in surface waters and highlights groundwater as an important but less visible exposure compartment.

More broadly, replacement flame retardants, organophosphate additives, UV filters, pharmaceuticals, and steroid hormones are routinely detected in effluents and receiving waters, typically from ng L^−1^ to low µg L^−1^ levels, with UV filters occasionally reaching µg L^−1^ concentrations in heavily impacted recreational waters. Although many of these compounds occur at low dissolved concentrations, their biological relevance may still be substantial, particularly when parent compounds and transformation products co-occur [60].

A comparative European monitoring study further demonstrated the geographic variability and cumulative risk potential of multi-class endocrine-disrupting contaminants in urban rivers. Using a harmonized SPE–LC–MS/MS method, 26 EDCs were quantified in the R. Liffey, R. Thames, and R. Ter over a 10-week campaign. Maximum concentrations reached 4767 ng L^−1^ for tris(2-chloroethyl) phosphate (TCEP) in the Thames, while cumulative concentrations approached ~2000 ng L^−1^ in London compared with ~543 and ~436 ng L^−1^ in the Liffey and Ter, respectively. Caffeine, benzotriazole, and organophosphate flame retardants exhibited high detection frequencies, whereas steroid hormones generally remained in the low ng L^−1^ range. Relative risk quotient analysis identified caffeine and BPA as consistent high-risk contributors across sites, indicating that mixture-driven ecological risk depends not only on absolute concentration, but also on compound class and urban pressure [10].

This mixture perspective is reinforced at the global scale. A recent assessment across 60 countries compiled 4489 country-level mean concentrations covering 190 PPCPs, of which 184 were detected above MDL in at least one country. Concentrations ranged from 0.01 ng L^−1^ for estriol to 94,307 ng L^−1^ for lamivudine, with a median concentration of 23.92 ng L^−1^ and cumulative national concentrations reaching 159.5 µg L^−1^ in Kenya. Ecological risk quotients identified hormones, NSAIDs, and antiepileptics as dominant contributors, accounting for more than 50% of total RQ in 54 countries, while ibuprofen, 17β-estradiol, carbamazepine, and estrone emerged as critical priority compounds [84]. Compared with the European river study, this global synthesis shows that mixture-driven prioritization is broadly consistent, although the magnitude of occurrence and risk remains strongly modulated by infrastructure, socioeconomic conditions, and hydrological dilution.

Coastal systems provide an additional perspective by integrating dissolved, particulate, and food-web processes. In the Bohai Sea, ΣPPCPs in seawater ranged from 57.5 to 309 ng L^−1^ across 25 stations, decreasing from coastal and estuarine zones toward the central basin, consistent with terrestrial and riverine inputs. Surface sediments contained 0.38–32.2 ng g^−1^ dw, while 53 PPCPs were detected in seawater and 40 in sediments. Antioxidants, bisphenols, and analgesic/anti-inflammatory drugs dominated seawater profiles, with benzotriazole reaching 109 ng L^−1^ [85]. In contrast to river monitoring focused mainly on dissolved concentrations, trophic magnification analysis in benthic food webs showed that ionization-corrected hydrophobicity (log Dow), rather than conventional log Kow, better predicted marine bioaccumulation.

High-throughput watershed monitoring highlights similar mixture complexity at the regional scale. In the Jingmi Water Diversion Canal (Beijing), a UPLC–MS/MS method targeting 323 PPCPs and pesticides detected 103 compounds, with antibiotics accounting for 25.2% of total detections. Mean total concentrations were significantly higher in winter (69.0 ng L^−1^; range 0–1746.4 ng L^−1^) than in summer (42.1 ng L^−1^; range 0–389.3 ng L^−1^), and urban sites consistently showed higher burdens than suburban sites [6]. Frequently detected compounds included caffeine, carbendazim, atrazine, diazepam, and desethyl atrazine, with detection frequencies up to 97.8%. Compared with the global assessment, this watershed-scale study shows that localized urban catchments can display similarly high mixture complexity, but with stronger seasonal amplification and sharper spatial contrasts [6,84].

Human biomonitoring and biota data further demonstrate that its occurrence in water can translate into internal exposure and bioaccumulation. In the SELF cohort, urinary concentrations of 16 phthalate metabolites, 7 phenols, 4 parabens, and triclocarban were quantified using online SPE coupled to isotope-dilution LC–MS/MS, with MDLs of 0.2–1.2 ng mL^−1^ for phthalates, 0.1–1.0 ng mL^−1^ for parabens, and 0.1–1.7 ng mL^−1^ for phenols. Principal component analysis identified mixture profiles dominated by phthalates, phenols, and parabens, with clear socioeconomic modulation of exposure patterns [86]. Likewise, in a national German fish survey downstream of WWTP discharges, only two pharmaceuticals were detected at low ng g^−1^ wet-weight levels, whereas synthetic musks reached much higher concentrations, up to 11,100 ng g^−1^ lipid weight for galaxolide [87]. Compared with dissolved-phase monitoring alone, these data show that lipophilic PCPs may exhibit much stronger bioaccumulation potential than many pharmaceuticals.

Finally, molecular EC occurrence is not restricted to surface waters, but also extends to groundwater and to fluorinated chemical space that remains incompletely characterized. In the first large-scale assessment across 18 U.S. Principal Aquifers (>1000 sites), at least one compound was detected at 5.9% of public-supply sites and 11.3% of domestic-supply sites, with 34 distinct compounds identified overall, including bisphenol A, carbamazepine, sulfamethoxazole, meprobamate, and 1,7-dimethylxanthine. Although national detection frequencies were modest, vulnerability was higher in shallow wells and under conditions favoring rapid transport and limited attenuation [88]. At the same time, a multistrategy target and non-target HRMS investigation in the Liuyang River watershed identified 106 fluorinated compounds across surface waters and municipal effluents, including 36 confirmed PFAS and 23 compounds not previously reported in environmental matrices [25]. Target PFAS concentrations ranged from 2.3–45.3 ng L^−1^ in winter surface waters and 6.1–65.6 ng L^−1^ in WWTP effluents, with short-chain PFCAs and PFSAs dominating [25]. These observations show that groundwater occurrence and fluorinated chemical diversity remain important but still under-characterized components of molecular EC monitoring, particularly where short-chain PFAS and non-target fluorinated compounds dominate.

Overall, the occurrence evidence reviewed in this section shows that molecular EC monitoring must move beyond single-compound and single-matrix assessments. Key trends include the persistence of WWTP and landfill inputs, the growing importance of reuse-driven soil–water–crop pathways, episodic stormwater pulses of tire-derived chemicals, and the increasing relevance of short-chain PFAS, transformation products, and non-target fluorinated compounds. However, inconsistencies among studies remain substantial because of differences in sampling design, target analyte lists, matrix coverage, analytical sensitivity, and reporting units. From a monitoring and regulatory perspective, these findings support the need for harmonized multi-matrix surveillance, event-responsive sampling for runoff-driven contaminants, and complementary target, suspect, and non-target workflows for contaminants that are not adequately captured by conventional priority lists.

### 2.2. Particulate Emerging Contaminants (Microplastics)

In aquatic systems, particulate emerging contaminants are dominated by MPs, including conventional polymer fragments and traffic-derived tire-wear particles. In contrast to molecular ECs, their environmental relevance depends not only on concentration, but also on particle size, polymer composition, surface properties, and their potential role as carriers of sorbed chemicals and microorganisms, which depends strongly on polymer type, environmental conditions, contaminant properties, and exposure scenario.

Experimental evidence indicates that MPs can act as polymer-specific sorbents for cyanotoxins, with adsorption governed by intrinsic material properties rather than simple passive partitioning. Among the polymers tested, polystyrene (PS) consistently showed the highest sorption affinity, followed by PE and PVC, whereas PET exhibited minimal adsorption under comparable conditions. Sorption capacity was linked to polymer physicochemical properties, including glass transition temperature, amorphous-domain content, and surface morphology, while smaller particles (0.09–0.125 mm) showed enhanced adsorption because of their larger reactive surface area. Adsorption also varied between toxin congeners, reflecting differences in hydrophobicity and pH-dependent speciation. Together, these results show that MPs are not inert particles, but dynamic phases that can influence the partitioning and transport of molecular contaminants [35].

More broadly, MP–EC interactions are controlled by both particle properties and environmental conditions. Recent reviews on pharmaceuticals and other emerging contaminants adsorbed onto MPs show that sorption involves several EC classes, including pharmaceuticals, PPCPs, pesticides, heavy metals, dyes, and natural organic matter, and different MP polymers such as PE, PP, PS, PET, PVC, PA, and biodegradable polymers. Sorption depends on polymer type, surface area, crystallinity, aging, surface functional groups, biofouling, and particle size, while contaminant properties such as hydrophobicity, charge, molecular size, and ionization state determine affinity for plastic surfaces. Field and in situ studies have detected pharmaceuticals such as atenolol, ibuprofen, sulfamethoxazole, ketoprofen, acetaminophen, carbamazepine, trimethoprim, erythromycin, and venlafaxine on plastic particles, with reported concentrations from ng g^−1^ levels up to approximately 1831 ng g^−1^ for acetaminophen on plastic litter. Adsorption studies for broader EC classes also show strong polymer- and contaminant-specific variability, including PPCP adsorption on PE, PP, PS, PVC, PET, PA, and biodegradable particles across µm–mm size ranges. Dominant mechanisms include electrostatic interactions, hydrogen bonding, hydrophobic partitioning, π–π interactions, cation bridging, van der Waals forces, pore filling, and partitioning into the polymer matrix. Environmental factors, including pH, salinity, ionic strength, dissolved organic matter, temperature, and competing natural particles, can further modify sorption and desorption behavior. Biofilm formation is particularly important because it changes MP surface polarity, roughness, functional-group availability, and mass-transfer kinetics, thereby either enhancing or slowing contaminant adsorption. Therefore, the vector role of MPs should not be interpreted as universal: in some settings MPs may enhance contaminant transport, persistence, bioavailability, or trophic transfer, whereas in others they may act mainly as temporary sinks or contribute less than natural organic matter and suspended solids. Field-based evidence remains limited, and many laboratory studies still use simplified systems, high concentrations, single polymers, or single contaminants. This highlights the need for environmentally realistic experiments, field monitoring, and combined chemical–particle analytical workflows to assess MP-associated EC risks more reliably [89,90].

Beyond sorption processes, plastic debris can also function as biologically active microhabitats. Greenhouse polyethylene films supported a distinct plastisphere and harbored 295 ARGs, including high-risk genes, with evidence of ARG–mobile genetic element (MGE) coupling. As opposed to cyanotoxin sorption, which is driven mainly by polymer properties, resistance enrichment on plastics appears to be governed by microbial colonization and selective antibiotic exposure. This comparison highlights the potential dual role of plastics as physicochemical sorbents and biologically active microhabitats, extending their relevance beyond contaminant partitioning alone [36].

Urban stormwater runoff is a major land-based pathway for particulate ECs, mainly tire-wear particles (TWPs). In Queensland, Australia, stormwater MPs > 25 μm ranged from 3.8 to 59 MPs L^−1^ in raw inlet water and from 1.8 to 32 MPs L^−1^ after treatment with a microlitter capture device, corresponding to 35–88% removal. TWPs accounted for about 95% of all particles, with concentrations of 2.5–58 TWPs L^−1^, indicating clear traffic-related dominance in urban runoff. Compared with the polymer-specific interactions described above, this case emphasizes source strength and hydrological transport as the dominant controls on particulate occurrence in stormwater systems [91].

WWTPs, in turn, represent major sinks for MPs, but not necessarily final barriers. Most plants operating at secondary treatment or above remove more than 78% of influent MPs from the water phase into sludge. However, when biosolids are applied to land, this captured load is redistributed rather than eliminated; in Europe, land application has been estimated to return about 125–850 tonnes of MPs per million inhabitants per year. Across compiled datasets, median MP concentrations were 225 MPs kg^−1^ dw in control soils, compared with 1106 MPs kg^−1^ dw in sludge-amended soils, while stabilized sludge itself reached a median of 18,923 MPs kg^−1^ dw [32]. Thus, compared with stormwater systems where capture devices can reduce particle loads before discharge, WWTP “removal” often represents transfer from water to solids, shifting the contamination burden from aquatic to terrestrial compartments.

Overall, these findings show that the environmental significance of MPs extends beyond their abundance alone and depends on polymer-specific interactions, sorption–desorption behavior, biological colonization, particle size, weathering state, and redistribution across water, sludge, soils, and stormwater pathways. However, comparisons among studies remain difficult because MP–EC interactions are strongly influenced by polymer type, aging state, biofilm development, contaminant properties, matrix composition, and experimental design. These inconsistencies highlight the need for harmonized MP monitoring protocols, environmentally realistic sorption/desorption studies, and cautious regulatory interpretation of MPs as contaminant carriers, distinguishing true vector effects from simple co-occurrence, temporary sorption, or transfer between environmental compartments.

## 3. Sampling and Sample Preparation Strategies

### 3.1. Sampling Design and Representativeness

Sampling design is a cross-cutting determinant of data quality for both molecular ECs and MPs because measured concentrations depend strongly on hydrology, source type, temporal variability, particle transport, and matrix characteristics. Therefore, sampling strategies should be selected according to the monitoring objective: event-triggered or composite sampling for short-lived runoff pulses, passive sampling for time-integrated diffuse inputs, and repeated long-term sampling for persistent or seasonal contamination patterns.

For storm-responsive contaminants such as 6PPD-quinone (6PPDQ), temporally resolved sampling is essential because concentrations can increase rapidly during rainfall and may peak after rainfall onset. Single grab samples may therefore miss both maximum concentrations and exposure duration, making event-triggered or composite sampling more appropriate for tire-derived contaminants and other runoff-driven ECs [17,67]. A similar principle applies to particulate contaminants in stormwater, where representative MP and tire-wear particle monitoring requires sufficient sample volume, flow- or event-based collection, and separation of dissolved and particulate fractions [91,92].

Source-oriented solid sampling can further complement water monitoring when runoff alone does not capture the full congener space. For tire-derived contaminants, analysis of end-of-life tires and road dust can help identify parent additives, quinone transformation products, and related structural analogues that may not be fully represented in dissolved stormwater samples [93]. This combined approach is particularly useful for non-target screening, where source materials can support compound prioritization and interpretation of environmental features.

In contrast to storm-focused sampling, long-term routine monitoring is necessary for contaminants characterized by chronic or widespread baseline occurrence. Multi-year river monitoring and repeated WWTP effluent sampling can reveal persistent exposure patterns, seasonal variability, and treatment-related changes that are not captured by isolated sampling events [79,94]. Compared with event-driven strategies, these long-term designs are better suited to trend analysis and chronic exposure assessment.

Passive sampling provides a complementary time-integrative approach, particularly valuable for diffuse agricultural pollution and first-flush events. Chemcatcher and related passive samplers can integrate contaminant exposure over deployment periods, reduce the influence of short-term dilution variability, and improve spatial comparison of tributary or catchment contributions [10,95]. Compared with grab sampling, passive sampling is especially useful for low-level, non-point inputs where episodic pulses and changing hydrological conditions complicate interpretation.

Overall, sampling design should be aligned with contaminant behavior, matrix type, and monitoring purpose. Event-triggered and composite sampling are most appropriate for hydrologically driven pulses, passive sampling is advantageous for diffuse and time-integrated exposure, and repeated long-term monitoring remains essential for trend analysis and chronic exposure assessment. This structure emphasizes representativeness as a common requirement for both molecular and particulate contaminants rather than treating water sampling as a separate issue. This is particularly important for regulatory monitoring because inconsistent sampling frequency, timing, and sample type can lead to non-comparable exposure estimates and may underestimate short-lived but toxic contamination pulses.

### 3.2. Sample Preparation for Molecular Emerging Contaminants

Sample preparation for molecular ECs must be matched to both matrix complexity and analytical objective, since workflows optimized for trace-level quantification are not always appropriate for non-target discovery or particle-resolved characterization. In general, miniaturized extraction strategies are attractive for cleaner aqueous matrices, whereas solid, saline, lipid-rich, or highly complex samples require stronger enrichment, matrix disruption, or cleanup. To improve readability and provide a classified overview, the main sample-preparation strategies for molecular ECs are summarized in Table 1 according to applicable matrices, advantages, and limitations.

This classification shows that sample preparation should be selected according to matrix complexity, analyte polarity and hydrophobicity, expected concentration range, and analytical objective, including targeted quantification, suspect/non-target screening, transformation-product elucidation, or particle-resolved nanomaterial characterization. A representative miniaturized approach is the combination of solid–liquid extraction (SLE) and dispersive liquid–liquid microextraction (DLLME) coupled to LC–HRMS for lipophilic marine biotoxins. In seawater, direct DLLME of 12 mL using 450 μL chloroform and 500 μL methanol enabled effective preconcentration, whereas mussel samples required prior SLE before DLLME because of matrix complexity and the need for lipid-compatible extraction. This matrix-dependent loss of performance highlights the importance of recovery assessment, matrix-effect evaluation, and appropriate cleanup when miniaturized extraction is transferred from water to biotic tissues [96]. Thus, compared with conventional SPE, DLLME offers low solvent consumption and rapid enrichment, but its selectivity may be limited in lipid-rich biological matrices.

For complex seafood matrices, matrix solid-phase dispersion (MSPD) offers a more integrated alternative by combining extraction and cleanup in a single step. In a Portuguese coast surveillance study of fish muscle, liver, and bivalves, MSPD coupled to LC-QTOF and GC-QTOF HRMS enabled broad multiclass screening, with pharmaceuticals mainly detected by LC-QTOF and industrial chemicals by GC-QTOF [97]. Compared with DLLME, MSPD is less miniaturized but more practical for heterogeneous biota extracts, especially when broad HRMS screening rather than targeted quantification is the priority.

At the opposite end of the workflow spectrum, large-volume solid-phase extraction remains central for low-abundance unknowns in drinking water. In a non-target GC–HRMS investigation of alicyclic halocyclopentadienes, 5 L of source and finished drinking water were acidified to pH < 1 and extracted on sequential XAD-2 and DAX-8 resins, followed by ethyl acetate elution and evaporation to 200 μL. Acidification improved recovery of neutral and weakly acidic halogenated species, while dual-resin extraction broadened capture of structurally diverse DBPs contributing to the unknown TOX fraction. Relative to miniaturized extraction, this approach is more labor- and solvent-intensive, but it is better suited to structural elucidation of previously unreported DBP classes [19].

For hydrophobic PCPs in sediment and biota, extraction is driven largely by strong lipid and solid-phase partitioning. Conventional LC–MS/MS remains the dominant approach for trace-level PCP analysis across water, sediment, and biota, although electrospray ionization is prone to signal suppression and enhancement in complex matrices, requiring matrix-matched calibration or isotope-dilution correction [52]. For sediment and fish tissues, Soxhlet and pressurized liquid extraction (PLE) are commonly applied, followed by cleanup steps such as gel permeation chromatography and silica or Florisil adsorption. This is especially important for UV filters, whose log Kow values of about 4–8 promote strong lipid co-extraction. Reported sediment methods illustrate the importance of recovery assessment and cleanup efficiency, with benzotriazole UV stabilizer recoveries of 50.1–87.1% after matrix-adapted extraction and cleanup. Compared with simpler aqueous workflows, sediment and tissue preparation therefore requires more extensive cleanup to balance recovery and matrix removal [98].

Targeted multi-matrix workflows for ubiquitous semi-volatile contaminants further illustrate that contamination control is often as important as extraction efficiency. In a validated LC–MS/MS method for eleven phthalate diesters, aqueous samples were acidified to pH 2, filtered, and extracted by polymeric SPE, whereas soil and municipal wastes were treated by repeated acetonitrile ultrasonication followed by SPE cleanup. Recoveries of 70–98% were achieved, outperforming Soxhlet extraction while reducing solvent consumption. However, the method also required isotopically labeled standards, baked glassware, phthalate-free consumables, and systematic procedural blanks to control laboratory background. Thus, in contrast to analyte-specific extraction challenges alone, ubiquitous contaminants demand preparation strategies that also minimize artefactual contamination [88].

The same principle applies when temporal representativeness is critical. During a COVID-19 peak monitoring campaign, 24 h composite influent and effluent wastewater samples were collected hourly, acidified to pH 2, stored at 4 °C in the dark, and processed within 2 weeks by SPE prior to LC–HRMS analysis. This example shows that sample preparation begins at preservation and storage, and that standardized pretreatment can be essential for comparability when wastewater loads vary rapidly over time [20].

For strongly sorbed contaminants in heterogeneous wastes, homogenization and extraction become especially important. In aged municipal landfill refuse, freeze-drying, homogenization, and solvent extraction enabled the recovery of pharmaceuticals from a complex solid matrix, while strong refuse–leachate partitioning for quinolones emphasized the need for rigorous preparation when contaminants are embedded in waste-derived materials [99]. Compared with water samples, such solids require more aggressive homogenization and matrix disruption before chromatographic analysis is feasible.

For engineered nanomaterials, sample preparation differs fundamentally from molecular extraction because the goal is to preserve particle number, size, aggregation state, and transformation state rather than transfer analytes into solvent. Therefore, preparation must minimize artefactual aggregation, dissolution, or speciation changes during filtration, dilution, extraction, and storage. Particle-resolved workflows, including nanoparticle tracking analysis, single-particle ICP–MS, and field-flow fractionation coupled to ICP–MS, require matrix-dependent calibration and careful preservation of the particle mass distribution [100,101]. Pretreatment strategies such as ultrafiltration, ultracentrifugation, cloud-point extraction, and pyrophosphate-based extraction can support enrichment and separation, but their suitability depends strongly on matrix composition and the need to distinguish engineered nanoparticles from natural colloids [13]. Compared with bulk metal analysis, these particle-resolved approaches are essential for tracking aggregation, dissolution, and transformation during treatment and environmental aging.

PFAS sample preparation is highly matrix- and objective-dependent. For regulatory and targeted analysis, polymeric SPE coupled with LC–MS/MS remains the most widely used workflow for aqueous samples because it provides robust enrichment and good compatibility with standardized methods. However, PFAS analysis requires strict contamination control, including fluoropolymer-free materials, procedural blanks, isotope-labelled standards, and careful assessment of background fluorine. Matrix-specific adaptations are also needed: industrially impacted waters may require SPE workflows compatible with both target and suspect screening, whereas solid consumer-product matrices require solvent-based extraction to cover ionic and neutral PFAS. For highly saline samples, standard addition or matrix-matched calibration can improve quantification by compensating for ion-suppression and recovery effects [53,82,102,103,104].

Bulk fluorine metrics provide a complementary preparation and measurement strategy for samples where compound-resolved PFAS analysis may not capture the full fluorinated fraction. EPA Method 1621 determines adsorbable organic fluorine through adsorption onto granular activated carbon, combustion at ≥1000 °C, and fluoride quantification by ion chromatography, with recoveries of 45–147% and an MDL of 1.5 μg F L^−1^ [60]. Although it lacks molecular specificity, adsorbable organic fluorine (AOF) can support screening and mass-balance interpretation when used alongside compound-specific PFAS methods.

Overall, these examples show that sample preparation must be tailored not only to contaminant class and matrix complexity, but also to the analytical purpose itself. Miniaturized extraction is advantageous for clean aqueous matrices, large-volume enrichment is more appropriate for low-abundance unknowns, extensive cleanup is essential for solids and biota, and particle-resolved pretreatment is indispensable for nanomaterials. Thus, extraction scale, sorbent chemistry, cleanup intensity, and analytical resolution must all be aligned with whether the goal is compliance monitoring, quantitative multi-residue analysis, or structural elucidation of previously unrecognized ECs. Consequently, method selection should be reported transparently together with recoveries, matrix effects, blanks, and quality-control criteria to improve interstudy comparability and support regulatory confidence in occurrence data.

### 3.3. Sample Preparation for Particulate Emerging Contaminants (Microplastics)

**Figure 2:**
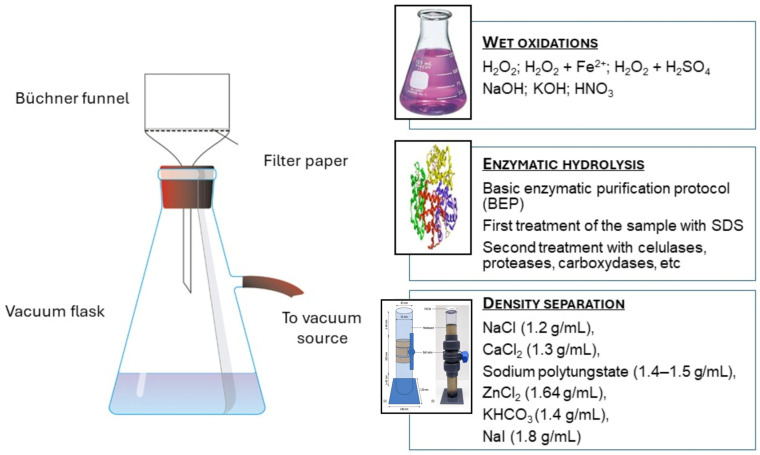
Representative extraction and clean-up workflow for MPs in complex environmental matrices.

Sample preparation for MPs and NPs must combine size-resolved isolation with effective removal of organic interferences while minimizing procedural contamination. Unlike dissolved molecular ECs, particulate workflows require a careful balance between sufficient matrix digestion and preservation of polymer integrity. As a result, most protocols follow a stepwise scheme involving particle capture, matrix cleanup, and density-based enrichment before spectroscopic or thermal identification. A schematic overview of these strategies is shown in Figure 2.

Common pretreatment strategies include wet oxidation for the removal of organic matter, enzymatic digestion for selective degradation of biological matrix components, and density separation for the enrichment of plastic particles prior to spectroscopic or thermal identification.

In wastewater matrices, one approach combined microfiltration/ultrafiltration with H_2_O_2_ digestion and sequential filtration on 1000, 50, and 1 µm stainless-steel membranes prior to pyrolysis–GC–MS analysis. This workflow enabled retention of both MP and NP fractions after oxidative cleanup, with total MP and NP mass concentrations decreasing from 26.2 and 11.3 µg L^−1^ in influent to 1.8 and 0.7 µg L^−1^ in effluent, corresponding to 93% and 94% removal, respectively [105]. Compared with particle-counting strategies, this example highlights the importance of high-throughput filtration combined with digestion when polymer-mass is the target metric.

For low-turbidity drinking or tap water, simpler front-end preparation may be sufficient. Direct size-selective capture using 25 µm stainless-steel filters mounted on taps, followed by micro-IR confirmation, reduced handling losses and background contamination and provided a defensible strategy when the target size window was clearly defined [106]. Thus, compared with wastewater, cleaner matrices may require less aggressive pretreatment and benefit from direct filtration approaches.

Solid environmental matrices such as soil, sediment, and sludge generally require more intensive dismantling to release embedded polymers before analysis. A protocol based on tetramethylammonium hydroxide digestion, ethanol washing to remove natural organic matter, and dichloromethane dissolution with ultrasonication achieved recoveries of 80–91% across PS, PE, PP, PMMA, PVC, and PET, with pyrolysis–GC–MS detection limits of 2.3–29.2 µg g^−1^ [60]. These results emphasize that extraction efficiency is strongly matrix- and polymer-dependent, making recovery validation essential for heterogeneous solids.

Tire road wear particles (TRWPs) require additional adaptation because their density (1.3–1.7 g cm^−3^) often exceeds the limits of conventional NaCl separation. A two-stage density separation using saturated NaCl (ρ ≈ 1.2 g cm^−3^) followed by sodium polytungstate (ρ = 1.9 g cm^−3^) recovered up to 98% of TRWPs below 1.9 g cm^−3^. Alkaline digestion with NaOH or KOH preserved microrubber structure, whereas H_2_O_2_ and NaOCl caused deformation. Because carbon black interferes with vibrational spectroscopy, confirmation required SEM and pyrolysis–GC–MS, with isolated TRWPs ranging from 63 to 500 µm [107]. Compared with conventional MP workflows, tire-derived particles therefore demand denser separation media and greater reliance on thermal or electron-based confirmation.

A similar issue arises in stormwater, where particle-rich matrices and dense fragments complicate recovery. A validated workflow combined filtration on 25 µm stainless-steel filters, oxidative digestion with 30% H_2_O_2_, high-density separation using NaI (1.8 g cm^−3^), and Rose-Bengal staining prior to stereomicroscopic screening of suspected MPs > 25 µm [91]. Compared with low-density separations alone, this approach is better suited for recovering TWPs and other dense anthropogenic particles that would otherwise be underestimated.

At the same time, newer analytical approaches aim to reduce the wet-chemistry burden while extending detection to smaller particles. IR photothermal heterodyne imaging has achieved a spatial resolution of about 300 nm with minimal sample preparation, and Raman spectroscopy combined with machine-learning classification reported 99% identification accuracy for NPs and >97% accuracy in spiked tap-water validation [60]. Compared with conventional digestion and density-separation workflows, these approaches shift selectivity toward instrumental resolution and chemometric classification, potentially reducing losses, contamination risks, and recovery bias associated with extensive pretreatment.

Overall, sample preparation for particulate ECs is more strongly constrained by particle properties than is the case for dissolved analytes. Wastewater and solid matrices generally require digestion and enrichment, drinking water can often be addressed by direct filtration, and tire-derived particles demand denser separation and alternative confirmation methods. However, inconsistencies in digestion efficiency, density separation, particle-size cut-off, and polymer-identification criteria continue to limit comparability among MP and NP studies. Therefore, harmonized QA/QC procedures, recovery validation, contamination control, and transparent reporting of size limits and polymer coverage are essential for reliable monitoring and future regulatory use of MP and NP occurrence data.

## 4. Analytical Strategies for Molecular Emerging Contaminants

The analytical determination of molecular ECs in aquatic environments requires methods that combine sensitivity, selectivity, and structural information across chemically diverse compounds occurring at trace levels. In this section, the discussion is organized according to analytical purpose rather than instrument type alone. Targeted LC–MS/MS workflows are first discussed as the quantitative backbone for predefined analytes and regulatory monitoring, with GC-based methods mentioned where relevant for specific contaminant classes. HRMS is then considered as a complementary platform that supports both expanded target confirmation and suspect/non-target screening, enabling the detection of transformation products, replacement compounds, and previously unrecognized contaminants. This organization clarifies that HRMS is not separate from target/non-target analysis, but rather provides the high-resolution acquisition and structural information needed to extend analytical coverage beyond predefined lists.

Because molecular ECs and MPs require fundamentally different analytical strategies, Table 2 provides a concise comparison of the main approaches discussed in this review. Detailed method-specific examples and references are provided in Supplementary Table S1.

### 4.1. Targeted LC-MS/MS Workflows

Targeted LC–MS/MS, typically based on triple-quadrupole instruments operating in multiple reaction monitoring mode, remains the reference technique for quantitative determination of priority ECs. Reliable application of these workflows requires matrix-effect evaluation, use of isotope-labelled or structurally similar internal standards, procedural blanks, recovery checks, calibration verification, and QA/QC criteria for ion-ratio confirmation and retention-time stability. Its main strengths are high sensitivity, excellent selectivity, and compatibility with interlaboratory standardization, generally allowing quantification from ng L^−1^ to sub-ng L^−1^ levels for predefined analytes.

Recent developments have expanded targeted LC–MS/MS toward chemically challenging classes such as PFAS. A multidimensional configuration integrating large-volume direct injection with dynamic heart-cutting between reversed-phase and HILIC–ion-exchange columns enabled simultaneous quantification of 60 PFAS without offline SPE, thereby improving coverage from ultrashort- to long-chain homologues. Matrix-specific LOQs were ≤1 ng L^−1^ for most analytes in surface and seawater, although trifluoroacetic acid showed a much higher LOQ (~500 ng L^−1^) because of background contamination [23]. Compared with conventional SPE–RP–LC–MS/MS workflows, this setup reduces extraction bias and broadens polarity coverage, while still remaining limited to predefined target compounds.

Regulatory standardization has further strengthened the role of targeted LC–MS/MS. EPA Method 1633 enables determination of 40 PFAS in aqueous, solid, biosolid, sediment, and tissue matrices using SPE–LC–MS/MS with isotopically labeled standards, achieving recoveries of 45–145% and LOQs of 1–100 ng L^−1^, most commonly 1–4 ng L^−1^ in water [60]. Similarly, Draft EPA Method 1634 for 6-PPDQ in water reports recoveries of 78–96% and an MDL of 0.43 ng L^−1^, whereas Draft EPA Method 1628 for PCB congeners uses low-resolution GC–MS with SIM and reports MDLs of 0.2–5.0 ng L^−1^ in water [60]. These developments show that targeted methods continue to expand toward newly recognized EC classes while maintaining strong regulatory comparability.

Beyond regulatory methods, targeted LC–MS/MS is increasingly adapted to difficult analytes and contamination-prone workflows. Phthalate diesters illustrate this well: a validated multi-matrix LC–MS/MS method quantified eleven phthalates in surface water, landfill leachate, soils, and municipal wastes with detection limits down to 0.2 ng L^−1^ and precision below 5% RSD [108]. Importantly, the workflow incorporated a chromatographic delay column to displace instrument-derived phthalate contamination, together with blank correction, isotopically labeled standards, and phthalate-free consumables. This example highlights that, for ubiquitous contaminants, analytical robustness depends as much on contamination control as on instrumental sensitivity.

Although the main focus of this review is water monitoring, selected multi-compartment and biomonitoring examples are included to illustrate how water-related contamination can be linked to uptake, bioaccumulation, and broader exposure assessment. Targeted LC–MS/MS also remains indispensable for multi-compartment exposure studies. In lysimeter-grown tomatoes irrigated with treated wastewater, validated UHPLC–MS/MS workflows using a hybrid triple-quadrupole/linear ion-trap platform combined with Oasis Prime HLB extraction were used to monitor 27 contaminants across irrigation water, soil, roots, leaves, stems, and fruits [38]. Under non-spiked irrigation, only carbamazepine was detected in fruits above LOQ (22 ng g^−1^), whereas up to 17 compounds accumulated in roots at ng g^−1^ levels; spiking experiments at 1 mg L^−1^ further confirmed compound-specific translocation to edible tissues. Similarly, a 21-day semi-static assay with Corbicula fluminea exposed to 6-PPD or 6-PPDQ used triple-quadrupole LC–MS/MS to verify water concentrations with an MDL of 0.01 µg L^−1^ for both analytes [109]. Such applications underline the continuing value of triple-quadrupole platforms for quantitatively linking external exposure to tissue-specific accumulation.

At the same time, targeted LC–MS/MS can now be scaled toward broader exposome-level panels. A next-generation human biomonitoring workflow using 96-well SPE and LC–MS/MS quantified more than 230 biomarkers across urine, plasma, and serum, with extraction recoveries and matrix effects largely within 60–130% and intraday/interday RSDs below 30%. LODs below 0.1 ng mL^−1^ were achieved for 59–80% of analytes, supporting high-throughput large-cohort studies [110]. Compared with classical priority-pollutant methods, such workflows expand analytical breadth substantially, although they remain confined to known and predefined targets.

This limitation becomes particularly evident for structurally diverse toxin families. In cyanotoxin analysis, more than 250 microcystin congeners have been described, yet routine targeted monitoring generally focuses on a small subset, often dominated by MC-LR [57]. The high-resolution analysis of algal dietary supplements revealed 35 additional minor congeners beyond those routinely targeted by LC–MS/MS, while bioassay-based methods such as ELISA and protein phosphatase inhibition assays often yielded higher equivalent concentrations because they captured cumulative biological activity rather than a restricted target list [111]. Thus, although targeted LC–MS/MS provides excellent sensitivity and structural specificity, it can systematically underestimate total toxin burden when congener diversity is high.

The same tension between quantitative robustness and restricted coverage is seen across other EC classes. Targeted LC–MS/MS using a triple-quadrupole platform has been used for long-term monitoring of 21 quaternary ammonium compounds in influent, effluent, and biosolids, achieving low ng L^−1^ detection limits in water and low µg kg^−1^ in solids while prioritizing homolog-specific surveillance over the expansion of chemical space [112]. Small, highly polar food-additive ECs such as artificial sweeteners have also been quantified by SPE–LC–MS/MS using negative electrospray ionization, with LODs of 0.006 mg L^−1^ and LOQs of 0.01 mg L^−1^ [53]. At the other end of the scale, nationwide U.S. river monitoring of imidacloprid across 12,547 samples from 77 sites used direct aqueous injection LC–MS/MS and showed detections in 44% of samples, with 39% exceeding the EPA chronic aquatic invertebrate benchmark of 10 ng L^−1^ [94]. Together, these examples show that triple-quadrupole methods remain highly versatile across EC classes, matrices, and monitoring scales.

Complementary rapid-screening tools can reduce analytical workload when full LC–MS/MS confirmation is not required. Multiplex competitive lateral flow immunoassays have enabled simultaneous detection of okadaic acid, saxitoxin, and domoic acid in shellfish with LODs of 0.1, 1.1, and 4.4 ng mL^−1^, respectively, and recoveries of 85.8–131.4% in spiked mussel extracts [113]. Electrochemical biosensors for cyanotoxins, particularly microcystin-LR, have reported exceptionally low detection limits in optimized nanocomposite designs and response times as short as 5–20 min in some saxitoxin platforms [114]. However, unlike LC–MS/MS, these methods generally provide limited structural confirmation and are therefore better viewed as rapid, semi-quantitative complements rather than replacements.

Overall, targeted LC–MS/MS remains the quantitative backbone of molecular EC analysis thanks to its sensitivity, selectivity, and regulatory compatibility. Its main limitation, however, is intrinsic restriction to predefined analyte panels. As contaminant space continues to expand through transformation products, replacement PFAS, low-abundance congeners, and uncharacterized toxins, targeted LC–MS/MS remains indispensable for robust quantification but increasingly requires support from HRMS, suspect screening, and effect-based tools to capture the full diversity of molecular ECs.

### 4.2. HRMS-Enabled Target, Suspect, and Non-Target Workflows

High-resolution mass spectrometry (HRMS), most commonly implemented as LC–HRMS or GC–HRMS on Orbitrap or QTOF platforms, supports both expanded target confirmation and suspect/non-target screening. Its value lies in accurate-mass measurement, full-scan acquisition, retrospective data analysis, and structural annotation of transformation products, replacement compounds, and previously unrecognized contaminants. Thus, HRMS should be viewed as a complementary platform that extends targeted LC–MS/MS and GC–MS/MS workflows rather than as a completely separate analytical category.

One of the key strengths of HRMS is its ability to combine quantitative determination with structural expansion. For example, DLLME coupled to LC–HRMS using a QTOF platform enabled simultaneous targeted quantification and suspect screening of eight regulated lipophilic marine biotoxins in seawater and mussel samples. Accurate-mass measurements within ±5 ppm, auto-MS/MS acquisition, and a suspect database containing 93 derivatives supported multidimensional annotation based on precursor mass, fragment ions, and mass-accuracy thresholds. Detection limits ranged from 0.0004–1.7 ng mL^−1^ in seawater and 0.06–119 ng g^−1^ in mussels, illustrating how HRMS can combine regulatory quantification with structural exploration of toxin derivatives in complex marine matrices [96]. A similar expansion of chemical space was demonstrated in Portuguese coastal biota, where MSPD extracts analyzed by LC–HRMS and GC–HRMS using QTOF platforms enabled tentative identification of 176 compounds and confirmation of 77 with standards, while also separating profiles according to tissue, sampling area, and campaign [97]. Compared with targeted workflows, these examples show the broader structural reach of HRMS in complex food-web relevant matrices.

HRMS is also particularly valuable for elucidating treatment-induced transformation products that lie outside predefined target lists. In acesulfame degradation experiments, UHPLC–MS/MS using a triple-quadrupole platform was used for kinetic monitoring, whereas UHPLC–HRMS using a QTOF platform identified chlorinated transformation products formed specifically under UV/monochloramine treatment. Under UV irradiation, 92% degradation was achieved, while monochloramine increased the apparent degradation rate and generated oxidant-specific products absent in UV-only systems [115]. Similarly, LC–HRMS suspect workflows coupled with transformation-product prediction and retention-time prediction enabled the identification of 114 pharmaceuticals and transformation products in WWTP samples during a COVID-19 peak, including 13 TPs not previously reported in wastewater [20]. HRMS has also proved valuable for tracking reactive intermediates and extending contaminant discovery to non-aqueous matrices. For example, UHPLC–HRMS using a QTOF platform was used to follow chlorinated intermediates formed from bisphenol analogues during disinfection, while complementary UHPLC–MS/MS quantified BDA formation, showing that BDAs formed preferentially under neutral conditions and were inhibited by chloramination, thereby illustrating disinfectant-dependent DBP formation pathways [116]. In parallel, LC–MS-based screening of aged municipal landfill refuse enabled quantification of 55 pharmaceuticals, of which 42 were detected at 0.30–116 μg kg^−1^, demonstrating that HRMS-compatible workflows can also extend contaminant profiling to complex solid waste reservoirs [99]. Together, these examples highlight a major contrast with triple-quadrupole methods: HRMS not only tracks parent removal, but also captures chemical space expansion during treatment and in secondary source matrices.

A further advantage of HRMS is its usefulness in mechanistic and source-diagnostic analysis. Compound-specific isotope analysis, typically performed by GC–IRMS or LC–IRMS, provides process-level evidence distinguishing dilution or sorption from true transformation by interpreting isotope fractionation of elements such as C, H, N, and Cl. Although not a conventional full-scan HRMS workflow, CSIA complements HRMS by clarifying reaction pathways in groundwater contamination, diffuse pesticide pollution, and water-treatment systems [117]. More generally, this illustrates that advanced mass-spectrometric strategies increasingly move beyond concentration data alone toward process interpretation. 

A major recent development has been the integration of ion mobility spectrometry (IMS) with HRMS. By providing collision cross section (CCS) values as an orthogonal structural descriptor alongside m/z, retention time, and MS/MS fragmentation, LC–IMS–HRMS improves the discrimination of structurally similar compounds, especially isomers. This is particularly relevant for PFAS, where high structural similarity often complicates confident annotation. Updated guidance now incorporates CCS evidence into standardized confidence levels for LC–IMS–HRMS or GC–IMS–HRMS identifications, thereby improving harmonization and transparency in PFAS reporting [118]. In environmental applications, UPLC–IMS–HRMS using a QTOF platform enabled annotation of 678 chemicals in estuarine sediments, including human and veterinary drugs (8.7%), food additives (6.5%), pesticides (2.8%), and PFAS (1.9%), while hotspot patterns reflected diffuse inputs and deposition zones [119]. Likewise, analysis of 156 U.S. serum samples by LC–IMS–HRMS using a QTOF platform combined accurate mass, retention time, and CCS matching to support the quantification of 19 PFAS and suspect screening of replacement compounds such as F-53B, 6:2 DiPAP, and chloroperfluorononylphosphonic acid [81]. Compared with LC–HRMS alone, the IMS dimension therefore improves structural discrimination and retrospective interpretation. 

HRMS has been especially transformative for PFAS analysis because it reveals how much fluorinated chemical space remains unresolved by target methods. Recent investigations identified 35 novel PFAS classes in commercial fluorinated products using UPLC–HRMS/MS on an Orbitrap platform, while newly discovered PFECAs accounted for 27–95% of total PFAS in industrial effluents, with individual compounds reaching 447 µg L^−1^ in effluent and 670 ng L^−1^ in nearby surface waters [120]. In drinking water, ultrashort-chain PFAS such as trifluoroacetic acid reached 12.4 µg L^−1^ and contributed up to 98% of total PFAS burden in some samples [121]. HRMS has also expanded PFAS profiling in complex solids: in e-waste recycling soils, LC–HRMS on an Orbitrap platform detected nine CF_3_-containing emerging PFAS beyond target panels, while TOP assay validation linked several of them directly to trifluoroacetic acid (TFA) formation [80]. Compared with predefined target workflows, these HRMS-based approaches reveal substantial precursor contributions and under-monitored short-chain species that strongly affect fluorine mass balance.

Complementary multidimensional resources further strengthen HRMS interpretation. An open collision cross-section (CCS) library containing 556 organic micropollutants measured in both ionization modes improved discrimination of isobaric and isomeric compounds and supported PFAS profiling in complex mixtures such as aqueous film-forming foams [122,123]. In parallel, ^19^F-NMR has emerged as a complementary technique for quantifying total fluorinated burden independently of chromatographic retention or ionization efficiency. Although its detection limits remain much higher than those of LC–MS methods and its structural resolution is limited in mixtures, ^19^F-NMR provides a matrix-independent measure of total PFAS that can contextualize LC–HRMS findings [124]. 

Finally, HRMS datasets gain additional power when combined with chemometric decomposition. In a passive-sampling campaign across three small streams and one river, full-scan LC–HRMS analysis on an Orbitrap platform followed by variance-based reduction and ASCA modeling separated spatial and temporal components of variation, while PLS-DA and volcano-based prioritization identified 223 site-specific and 45 season-specific discriminating features. Notably, the non-target dataset provided clearer separation of pollution fingerprints than target-only analysis, illustrating how HRMS transforms feature-rich data into ecologically interpretable contamination patterns [95].

Overall, HRMS extends analytical coverage far beyond predefined target panels by coupling accurate mass, full-scan acquisition, structural annotation, and retrospective analysis. Its main strengths lie in suspect and non-target screening, transformation-product discovery, PFAS structural expansion, and multidimensional workflows integrating IMS, chemometrics, or complementary total-fluorine approaches. Compared with targeted LC–MS/MS, HRMS offers substantially broader chemical-space coverage, although annotation confidence, data complexity, and the need for advanced processing remain important limitations.

### 4.3. Non-Target and Suspect Screening Approaches

Suspect screening (SS) and non-target screening (NTS) use full-scan LC–HRMS, GC–HRMS, or LC–IMS–HRMS datasets to prioritize compounds beyond predefined target lists. Their reliability depends on layered evidence, including accurate mass, isotopic pattern, MS/MS fragmentation, retention behavior, blank filtering, and, where available, collision cross section (CCS) values. Therefore, the key challenge is not only feature detection, but also transparent identification confidence and reproducible prioritization.

PFAS analysis provides one of the clearest demonstrations of the value of suspect and non-target workflows. In a European hotspot setting, combined target screening of 77 PFAS and suspect screening of about 120 additional PFAS using LC–HRMS on an Orbitrap platform expanded fluorinated chemical space well beyond standard drinking-water monitoring panels. Candidate suspects were extracted within ±10 ppm and filtered by blank subtraction and intensity thresholds, while retention-time homolog patterns, isomeric peak shapes, and HRMS/MS comparison with previously characterized AFFF formulations strengthened identification confidence [83]. This strategy revealed industrially consistent precursor and fluorotelomer signatures, including N-SPAmP-FHxSAA, bistriflimide, and fluorotelomer betaines/sulfonates, which would be missed by conventional Σ20 PFAS target lists [82].

Because PFAS structural diversity now spans thousands, and potentially far more, candidates, harmonized confidence frameworks have become essential. Updated PFAS-specific guidance proposes a five-level confidence system integrating accurate mass, MS/MS evidence, retention time, and CCS values, while emphasizing that tolerances for these parameters should remain instrument- and method-specific rather than universally fixed [118]. In practice, CCS reproducibility is generally good within a single IMS platform, but differences between technologies such as drift-tube and traveling-wave IMS require transparent reporting of platform-specific tolerances. Thus, compared with conventional suspect screening based only on mass and fragments, multidimensional frameworks can improve annotation consistency but remain limited by the availability of reliable CCS libraries.

To strengthen this multidimensional evidence layer, dedicated tools such as FluoroMatch IM have been developed. This vendor-neutral platform integrates CCS matching, molecular formula prediction, homologous series detection, mass-defect filtering, and accurate-mass database matching, thereby reducing false discovery rates in LC–IMS–HRMS datasets [125]. A broader example of fluorinated-compound screening was reported for an urban river system using integrated target and non-target LC–HRMS workflows. By combining target analysis of 45 PFAS with library matching against more than 7096 fluorinated compounds, homologous-series analysis (CF_2_, CF_2_O, CF_2_CH_2_, C_3_F_6_O series), molecular networking, analogue searching, and reaction-database-guided annotation, the workflow identified 106 fluorinated compounds across surface waters and municipal effluents, including 36 confirmed PFAS and 23 compounds not previously reported in environmental matrices [25]. Compared with CCS-focused PFAS workflows, this strategy relied more heavily on hierarchical evidence layering and spectral propagation to expand fluorinated chemical space while controlling false positives.

In contrast to these broad fluorinated workflows, marine toxin analysis illustrates a more constrained but highly structured form of suspect screening. A curated library of 93 toxin derivatives was integrated into a targeted feature-extraction workflow using predefined ±5 ppm mass tolerances and a minimum match score threshold of 80% [96]. This ensured transparent and reproducible filtering of candidate features, but the workflow remained inherently dependent on prior database inclusion. Compared with algorithm-assisted networking or IMS-enhanced PFAS workflows, such strategies emphasize controlled suspect expansion rather than open-ended discovery [96].

At the multiclass level, complementary LC–HRMS and GC–HRMS platforms have demonstrated the value of broad suspect screening for mixture characterization across environmental, food, and human matrices. In one large-scale study, pooled wastewater, fish, and human serum samples were analyzed using both LC–HRMS on an Orbitrap platform and GC–HRMS, and spectral matching against MoNA and MassBank enabled annotation of 547 compounds at Schymanski confidence levels 1–3, including 63 confirmed at Level 1. Wastewater showed the highest chemical diversity (341 compounds), whereas fish samples contained 25–27 detected compounds. The combined use of LC and GC platforms expanded chemical space coverage substantially, achieving about 99% detectability of QA/QC compounds when both techniques were applied together [126]. Compared with PFAS-specific IMS-supported workflows, this approach prioritizes broad mixture coverage rather than fine structural discrimination of closely related isomers.

A complementary hybrid strategy combines total fluorine screening with compound-specific confirmation. In reusable feminine hygiene products, particle-induced gamma emission identified intentional fluorination in 33% of period underwear and 25% of reusable pads, after which LC–MS/MS and GC–MS-based workflows confirmed PFAS in all extracted samples, with 6:2 and 8:2 fluorotelomer alcohols dominating. Products classified as non-intentionally fluorinated contained Σ42PFAS of 21–880 ng g^−1^, whereas intentionally fluorinated products contained 48–2205 ng g^−1^ [104]. This workflow illustrates how broad elemental-level screening can prioritize suspect materials, while compound-resolved mass spectrometry provides molecular confirmation and semi-quantitative assessment.

Another large-scale application of combined target and LC–HRMS non-target analysis was demonstrated in longitudinal monitoring of two major German river systems. In contrast to class-specific PFAS workflows or broad multiclass LC–GC suspect screening, this study combined quantitative LC–MS/MS with LC–HRMS on an Orbitrap platform and feature-based molecular networking to examine mixture evolution along urban gradients. Rather than emphasizing annotation counts alone, the workflow linked chemical features to catchment characteristics such as wastewater input and land cover, while molecular networking propagated structural information to spectrally related but previously unannotated features [127]. This example shows that large-scale NTS can support both compound discovery and source attribution, and longitudinal mixture interpretation.

Similar SS/NTS logic has been applied to less studied contaminant classes in remote systems. In Arctic soils and sediments, target and suspect screening of polyhalogenated carbazoles (PHCZs) revealed Σ11PHCZ concentrations of 0.06–165.6 ng g^−1^ dw in soils and 1.2–4.5 ng g^−1^ dw in sediments, with 100% detection frequency [128]. These findings confirm that dioxin-like emerging contaminants can occur even in remote environments and illustrate how SS/NTS approaches expand analytical scope beyond conventional POP monitoring frameworks.

The same expansion is increasingly important for solid environmental matrices. In sewage sludge, nanoLC–HRMS using an Orbitrap platform combined with semi-quantitative modeling enabled broad contaminant profiling in biosolids, linking Schymanski-based structural confirmation with isotopically assisted response-factor estimation [129,130]. Compared with water-based multiclass screening, these workflows place greater emphasis on sensitivity and matrix-adapted extraction, but still face uncertainty arising from semi-quantification and chromatographic bias toward moderately hydrophobic compounds [130].

Non-target workflows have also become more selective through reactivity-driven prioritization. A recent approach combining stable-isotope labeling with glutathione probes and HPLC–HRMS screened chlorinated and chloraminated waters for electrophilic DBPs, reducing >50,000 raw features to 255 isotopically paired adducts and ultimately identifying 202 DBPs, including 193 newly reported compounds. Mechanistic grouping further distinguished chlorinated substitution products from unsaturated addition products. Compared with broad multiclass suspect screening, this strategy narrows chemical space toward biologically reactive contaminants, increasing toxicological relevance, although it remains limited to probe-reactive species and is not inherently quantitative [131].

GC–HRMS has likewise expanded non-target analysis for volatile and halogen-rich DBPs. In chlorinated and chloraminated waters, high-resolution EI-TOF-MS combined with 5 L XAD-resin enrichment enabled identification of alicyclic HCPDs through accurate mass and characteristic Cl/Br isotope patterns, with six compounds detected exclusively after disinfection. Complementary GC × GC improved isomer separation, although Level 1 confirmation still required authentic standards. Compared with LC–HRMS suspect workflows, this GC–HRMS configuration is better suited to volatile DBPs contributing to the unresolved TOX fraction [19]. 

The scale of this unresolved DBP chemical space is illustrated by recent curation efforts. A comprehensive database compiling 6310 DBPs reported between 1974 and 2022 classified only 651 as confirmed, whereas 1478 were identified and 4142 remained proposed structures. This imbalance highlights a central limitation of SS/NTS workflows: full-scan acquisition greatly expands candidate discovery, but exposure-relevant interpretation remains constrained by standard availability, confidence harmonization, and transferability from laboratory formation studies to real environmental samples [4].

Effect-directed analysis (EDA) coupled with HRMS has been especially powerful for toxicant discovery. The identification of 6-PPDQ as the driver of coho salmon mortality is a well-known example, linking HRMS-based structural elucidation to an acute LC_50_ of 95 ng L^−1^ and subsequent urban stormwater concentrations up to 2.3 µg L^−1^ [60]. Wide-scope HRMS screening of snowmelt similarly detected 489 chemicals, including tire-derived transformation products from 1.3 ng L^−1^ to 75 µg L^−1^, highlighting transient pulses that targeted analysis might overlook [132]. More recently, a fragmentation-pattern-based HRMS workflow using diagnostic ions and neutral-loss criteria enabled recognition of six known and three previously unreported PPD-quinones across tire tissue, PM_2.5_, and surface soils, including first quantification of 8PPD-Q and 66PD-Q. Compared with earlier suspect-screening strategies, this diagnostic-fragment approach improves structural confidence for rubber-derived transformation products lacking commercial standards [26]. A related development is the integration of bioassay guidance into suspect and non-target workflows. In drinking water, virtual effect-directed analysis (vEDA) coupled LC–HRMS and GC–HRMS datasets with ERα-CALUX bioassays to prioritize features associated with observed biological activity rather than structural plausibility alone [133]. Similarly, in cyanotoxin analysis, targeted LC–MS/MS, LC–HRMS, ELISA, and PPIA were combined within a single workflow, showing that effect-based assays often captured broader cumulative toxicity than congener-specific quantification alone [111]. Compared with purely structure-driven SS/NTS, such hybrid strategies better support toxicological completeness and mixture-level interpretation.

Large-scale sludge screening further illustrates the scalability of HRMS for solid matrices. A Swiss nationwide sludge survey combined multi-extraction strategies with LC–HRMS on an Orbitrap platform and used homologous-series logic and Kendrick mass defect analysis to expand the annotation of surfactants and PFAS beyond library-confirmed compounds. Compared with water-based screening, these sludge workflows are more oriented toward integrative exposure assessment, mass-load estimation, and transformation dynamics during anaerobic digestion [28].

Algorithmic refinement is also becoming central to transformation-product discovery. Entropy similarity–driven transformation reaction molecular networking (ESTRMN) integrates LC–HRMS data acquired on an Orbitrap platform with entropy-based spectral similarity scoring and transformation knowledge-guided prediction to resolve parent–product relationships in wastewater. Compared with broad multiclass suspect screening or CCS-supported PFAS workflows, this strategy is more explicitly reaction-aware and aims to improve mechanistic interpretation of TP formation [27].

A similar class-oriented advance has been demonstrated for ladder polyether marine toxins, where fragmentation-rule–based screening of HRMS/MS data exploited sequential dehydration and desulfation patterns together with family-specific diagnostic fragments to classify polyether-like features prior to database matching [134]. Compared with conventional library-dependent NTS, such rule-based workflows enable faster class-specific toxin discovery, although unambiguous distinction of structural isomers still requires orthogonal evidence.

Despite these advances, identification confidence in SS/NTS workflows remains strongly dependent on spectral-library completeness, high-quality MS/MS reference spectra, and transparent reporting of acquisition parameters and tolerance criteria.

Overall, SS and NTS workflows substantially expand analytical coverage beyond conventional target lists by enabling the discovery of previously unmonitored compounds, transformation products, and mixture patterns across water, biota, sludge, and consumer-product matrices. Their main strengths lie in chemical-space expansion, retrospective data interrogation, and increasingly refined prioritization through CCS support, molecular networking, reactivity-directed screening, and bioassay integration. However, these advantages remain constrained by annotation uncertainty and limited reference-standard availability, making harmonized confidence assignment essential for translating expanded feature detection into reproducible and risk-relevant environmental interpretation.

Table S1 comparatively summarizes the principal analytical configurations currently applied to molecular ECs, including targeted LC–MS/MS, multidimensional LC–IMS–HRMS, and suspect/non-target screening workflows, with emphasis on instrumentation, analytical performance, identification capability, major strengths, and key methodological limitations.

## 5. Analytical Strategies for Particulate Emerging Contaminants (Microplastics)

### Spectroscopic and Thermal-Mass-Based Techniques

Spectroscopic techniques remain central to microplastic (MP) analysis because they preserve particle-level information, including size, shape, and polymer identity. Their main strength is therefore not only polymer assignment, but also morphological characterization, which is essential for interpreting transport, weathering, and potential biological interactions. Recent developments increasingly combine imaging with vibrational spectroscopy to extend analysis toward smaller particles. For example, nanoplastics released during repeated uncapping and recapping of plastic water bottles were characterized by SEM, single-particle extinction and scattering (SPES), and μ-Raman spectroscopy, revealing releases on the order of a few tenths of ng per opening/closing event and particles dominated by amorphous polyethylene, likely derived from cap abrasion. Similarly, in tap-water monitoring, direct collection on 25 μm stainless-steel filters followed by micro-IR spectroscopy enabled particle-resolved identification, with mean concentrations of 12.5 MPs m^−3^ and 32.2 anthropogenic particles m^−3^; the dominant polymers were polyamide, polyester, and polypropylene, with a lower contribution from poly(lactic acid) [60]. Despite these strengths, vibrational methods remain limited for the lower nanoplastic range by size-dependent detectability, spectral interferences, and relatively low throughput.

**Figure 3:**
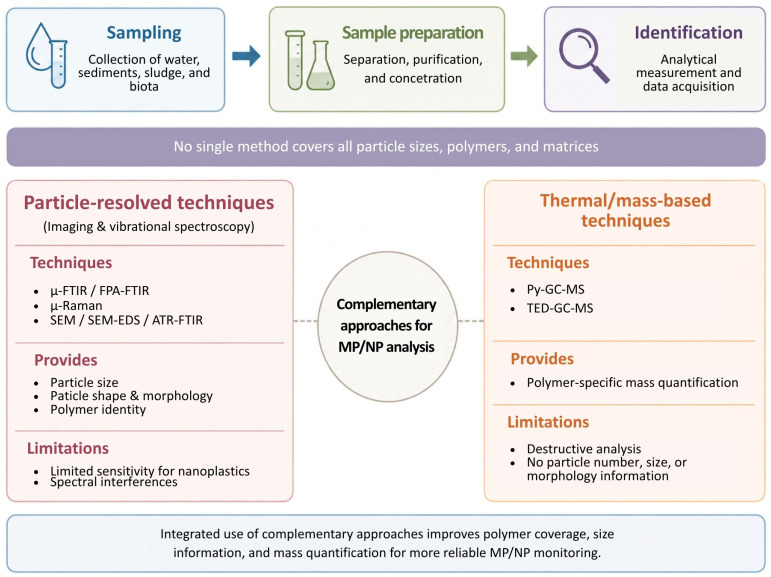
Complementary analytical approaches for microplastic characterization.

Because the information delivered by MP methods is inherently complementary, no single platform currently provides complete characterization across all particle-size classes, polymer types, and environmental matrices. Imaging and vibrational spectroscopy provide particle-resolved information, whereas thermal and mass-based methods provide polymer-specific bulk quantification. A comparative overview of these complementary analytical windows is presented in Figure 3.

For practical method selection, key performance parameters should be reported together with MP abundance or mass data. For spectroscopic methods, this includes the minimum detectable particle size, spectral-match criteria, and the polymer library used, because µ-FTIR is generally applied to low-micrometre particles whereas µ-Raman can target smaller MPs and selected NPs but is more sensitive to fluorescence, mapping time, and thermal damage. For thermal-mass-based methods, polymer coverage depends on the availability of specific pyrolysis markers and calibration standards; commonly quantified polymers include PE, PP, PS, PET, PVC, PMMA, PA, PC, ABS, SBR, PUR, and selected biodegradable polymers such as PLA, PCL, PBS, PBAT, and PHAs. Reliable MP detection also requires strict QA/QC, including procedural and field blanks, clean or covered handling, non-plastic or pre-rinsed materials, recovery tests with representative polymers and size classes, explicit reporting of size cut-offs, and confirmatory analysis for optically ambiguous, black, or weathered particles.

Thermal and mass-based methods, particularly pyrolysis–GC–MS and related thermal–MS workflows, complement spectroscopic techniques by providing destructive but polymer-specific mass quantification. In contrast to vibrational methods, which emphasize particle counts and morphology, pyrolysis–GC–MS identifies polymers through characteristic degradation products and is therefore especially useful for nanoplastics, highly weathered particles, and optically ambiguous materials such as tire wear particles (TWPs). In urban stormwater, coupling particle counting with confirmatory thermal/MS enabled more robust characterization of TWPs: outlet concentrations after treatment were 1.8–32 MPs L^−1^, including 1.3–32 TWPs L^−1^, corresponding to 0.2–1.1 mg L^−1^ TWPs [91]. In aqueous matrices, ultrafiltration combined with H_2_O_2_ digestion before pyrolysis–GC–MS enabled quantification of six polymers (PVC, PMMA, PP, PS, PE, PET), with linear calibration (R^2^ ≥ 0.98) and total nanoplastic concentrations up to 0.79 µg L^−1^ in surface water and 0.20 µg L^−1^ in groundwater [135]. Thermal/MS has also been extended to biodegradable polymers: pressurized liquid extraction followed by pyrolysis enabled quantification of PLA, PHA, PBS, PCL, and PBAT with recoveries of 74–116% and LOQs of 0.02–0.05 mg g^−1^ [136]. In soils, sediments, and sludge, recoveries of 79.6–91.4% were reported for PS, PE, PP, PMMA, PVC, and PET, with detection limits of 2.3–29.2 µg g^−1^ and total environmental concentrations of 4.6–51.4 µg g^−1^ [137]. Sensitivity has been further improved by coupling pyrolysis to triple-quadrupole MS in dynamic MRM mode, allowing quantification of twelve polymers at nanogram levels, with polymer-specific LODs as low as 1 ng and concentrations ranging from 2–35 µg L^−1^ in surface waters, 6.9–185.9 µg g^−1^ (dw) in sediments, and 0.1–13.3 mg g^−1^ (dw) in biota [138].

Overall, spectroscopic and thermal-mass-based methods should be regarded as complementary rather than competing strategies. Spectroscopic workflows provide particle-resolved identity and morphology, whereas thermal methods provide polymer-resolved bulk mass independent of optical detectability. Because thermal methods are destructive and do not preserve particle number, size, or shape, they are best applied as orthogonal mass-based tools alongside vibrational imaging when robust interpretation requires reconciliation of particle abundance with polymer load.

Table 3 comparatively summarizes the principal analytical strategies currently applied to particulate emerging contaminants (microplastics), highlighting their instrumentation, analytical outputs, main strengths, and key limitations.

## 6. Environmental and Human Health Risk Assessment

To improve readability and link analytical evidence with risk interpretation, this section is organized around four complementary risk-assessment dimensions: risk characterization based on occurrence, toxicity thresholds, risk quotients (RQ), and predicted no-effect concentrations (PNEC); transformation products, mixture effects, and treatment-driven toxicity; the role of MPs as contaminant carriers and modifiers of bioavailability; and human exposure pathways through drinking water, reuse systems, food webs, and biomonitoring. This framework emphasizes that risk assessment depends not only on measured concentrations, but also on analytical coverage, matrix effects, transformation processes, particulate-bound fractions, and uncertainty in target and non-target identification. The main risk indicators and their relevance for EC and MP assessment are summarized in Table 4.

### 6.1. Risk Characterization, Analytical Uncertainty, and Environmental Risks of Molecular ECs

Environmental risks of molecular emerging contaminants are governed not only by their occurrence in water systems, but also by transformation processes, chronic emission pathways, bioavailability, and species-specific sensitivity. In many cases, treatment or environmental transformation does not eliminate risk, but instead reshapes it through the formation of more reactive, toxic, or persistent products. Therefore, risk assessment should be interpreted together with analytical coverage, because incomplete target lists, lack of authentic standards, matrix effects, particulate-bound fractions, and uncertain HRMS annotations can all influence which contaminants are detected, quantified, and prioritized. Accordingly, risk assessment must integrate parent compounds, transformation products, transmissible biological hazards, and matrix-dependent exposure conditions.

Transformation processes can modify both the persistence and hazard of molecular ECs. For fluorinated contaminants, confirmed environmental defluorination remains limited, although mechanistic enzymatic studies demonstrate that C–F bond cleavage can occur under specific biochemical conditions [139]. However, for PFAS, environmental risk is still dominated by persistence, mobility, precursor transformation, and sustained emissions rather than by demonstrated natural degradation. Life-cycle and emission studies show that substitution from legacy to emerging PFAS may change the composition of fluorinated contamination without necessarily reducing the total burden entering air, water, soils, and wastewater systems [78,140]. This creates an analytical and regulatory challenge because many replacement PFAS, precursors, and ultrashort-chain species are not fully captured by conventional target panels. 

WWTPs further illustrate why apparent removal should not be interpreted as risk elimination. Biological treatment can transform PFAS precursors and may increase the effluent concentrations of some terminal PFAAs, while low ng L^−1^ effluent concentrations can still produce substantial cumulative loads when discharges are continuous [79]. Thus, WWTPs should be considered redistribution and transformation nodes rather than complete barriers, reinforcing the need to monitor both parent compounds and transformation products in influents, effluents, sludge, and receiving waters.

A similar logic applies to bioactive pharmaceuticals, illicit drugs, and antibiotic-resistance-related contaminants. Psychopharmaceuticals and drugs of abuse occur globally in surface waters, usually from pg L^−1^ to µg L^−1^ levels, and ecological risk can be enhanced in regions with limited wastewater treatment or low dilution capacity [141]. Photochemical transformation can either attenuate or increase hazard depending on matrix composition, dissolved organic matter, nitrate, pH, and irradiation conditions. For example, transformation of compounds such as cocaine or carbamazepine can be strongly matrix-dependent, and some transformation products may be more toxic than their parent compounds [21]. Therefore, risk assessment based only on parent-compound disappearance may underestimate residual toxicity.

Beyond chemical transformation, ARGs introduce a distinct environmental risk dimension because they behave as transmissible biological pollutants rather than conventional chemical toxicants. Landfills and insufficiently treated wastewaters can promote co-selection between ARGs, mobile genetic elements, metals, and antibiotic residues [40,44]. Mixture exposure is also important, because non-antibiotic pharmaceuticals can lower selection thresholds for resistance markers under combined exposure conditions [33]. This supports a more integrated risk-assessment framework in which chemical monitoring, biological markers, and mixture effects are interpreted together.

Engineered carbon nanomaterials represent another distinct class of molecular ECs whose risks extend beyond intrinsic toxicity to include vector and mixture-modulating effects. Under estuarine microcosm conditions, fullerene soot, multiwall carbon nanotubes, and graphene underwent aggregation and surface transformation that altered biological responses. Fullerene soot showed moderate intrinsic toxicity toward Vibrio fischeri (EC_50_ ≈ 26 mg L^−1^), whereas Daphnia magna was generally more sensitive. In binary mixtures with organic contaminants such as malathion, diuron, glyphosate, and triclosan, interactions were mostly antagonistic, although synergistic effects were observed in some cases, notably malathion–fullerene soot toward D. magna [142]. From an analytical perspective, this means that bulk metal or carbon-based concentration measurements alone are insufficient for risk assessment; particle-resolved methods are needed to distinguish engineered nanoparticles from natural colloids and to track transformations during treatment and environmental aging. Smaller aggregates were more toxic because of higher surface reactivity and bioavailability, and the nanomaterials also acted as vectors for co-contaminants, modifying internal exposure. Accordingly, nanomaterials should be considered not only as toxicants themselves, but also as modifiers of contaminant fate and mixture toxicity.

Treatment-driven transformation is also central to DBP and sweetener-related risks. Disinfection processes can reduce parent compounds while generating structurally distinct DBPs or transformation products with different toxicological profiles. Chlorination, chloramination, ozonation, and UV–chlor(am)ine processes can shift product distributions toward carbonaceous, nitrogenous, quinone-like, or chlorinated transformation products [115,116,143]. Similarly, UV-transformed sucralose showed markedly increased acute toxicity, with the EC_50_ decreasing from 2670 to 156.2 mg L^−1^ in Microtox assays [53]. The key risk message is therefore not only whether the parent contaminant is removed, but whether treatment generates products with higher persistence, reactivity, or biological activity. This highlights the need to consider transformation products in advanced treatment and reuse risk assessment, combining targeted analysis with HRMS-based suspect/non-target screening and, where possible, effect-based assays.

Rubber-derived transformation products provide a clear example of risk amplification after environmental transformation. 6PPD-quinone has been identified as a highly toxic tire-derived transformation product, with strong species-dependent effects in fish and particularly high sensitivity reported for some salmonids [68,144]. Sublethal effects, including oxidative stress, enzyme inhibition, and behavioral impairment, have also been observed in aquatic organisms exposed to 6PPD or 6PPD-quinone [109]. In addition, other rubber-derived transformation products, such as 4-hydroxydiphenylamine, may contribute endocrine activity in elastomer leachates [145]. These findings show that tire-derived risk cannot be evaluated from parent additives alone; transformation products, mixture toxicity, storm-driven exposure pulses, and species sensitivity must also be considered.

Plastic-associated additives from consumer products, including cigarette filters and other polymeric wastes, may contribute to environmental release of phthalates and related additives, but these pathways should be interpreted as secondary examples compared with the main water-linked sources discussed above [146]. 

At the management scale, the magnitude of tire-derived emissions supports treating these contaminants as a global pollution issue rather than a localized runoff problem. Global tire production reached 23 million tonnes in 2024, and wear-related losses correspond to estimated tire-wear particle emissions of 1.12 million tonnes yr^−1^ in the United States and 1.327 million tonnes yr^−1^ in the European Union. Because additives and fillers account for about 16–17% of tire mass, this represents a dispersed chemical load of roughly 185,000 tonnes yr^−1^ and 225,000 tonnes yr^−1^, respectively. In response, a management framework for tire additive pollution has been proposed that emphasizes safer alternatives, life-cycle assessment, transparency in tire composition, rigorous effects characterization, and international harmonization [147]. Upstream reformulation strategies, including bio-based elastomers, regenerated carbon black, rice-husk-derived silica, recycled polystyrene, and vegetable-oil plasticizers, may also reduce future emissions; however, their effects on additive release and transformation-product formation remain poorly characterized [148]. Together, these findings indicate that effective mitigation will require both downstream control and upstream redesign to avoid regrettable substitution.

Although this subsection focuses on molecular ECs, particulate contaminants can also modify the environmental risk profile of co-occurring chemicals. Micro- and nanoplastics (MNPs) can alter aquatic biogeochemical processes and contaminant behavior. A recent synthesis showed that MNPs interfere with carbon, nitrogen, sulfur, and phosphorus cycling by altering microbial community structure, enzyme activity, and redox microenvironments. In addition, plastisphere biofilms can act as reservoirs for pathogens and ARGs, thereby enhancing horizontal gene transfer and ecological persistence. MNPs also interact synergistically with other emerging contaminants through adsorption, hydrophobic partitioning, and electrostatic interactions, which may increase contaminant bioavailability and toxicity [34]. Thus, unlike conventional particulate carriers, MNPs function as active ecological modifiers that can indirectly amplify contaminant risks and water-security concerns.

For engineered nanomaterials, recent work has further shown that environmental risk cannot be assessed from environmental occurrence alone. A USEtox-based life-cycle assessment of carbon nanotubes indicated that aquatic ecotoxicity associated with production processes could equal or exceed impacts from modeled environmental CNT releases, with production-stage burdens largely driven by metal emissions from electricity generation [149]. This upstream perspective contrasts with monitoring-based studies focused only on post-release concentrations, and suggests that dominant ecotoxic impacts may arise before nanomaterials even enter aquatic systems. Accordingly, nanomaterial risk assessment should integrate emission inventories, fate processes, and ecotoxicity characterization within a life-cycle framework rather than relying solely on measured environmental concentrations.

Region-specific nanoscale fate modeling also highlights how strongly nanomaterial risk differs from that of dissolved contaminants. For copper nanoparticles, a freshwater USEtox-based model incorporating dissolution, aggregation, pseudosedimentation, advection, and resuspension produced characterization factors ranging from 3.87 × 10^3^ to 11.11 × 10^3^ CTUe across 17 subcontinental regions, with Africa identified as the most sensitive region. Sensitivity analysis showed that freshwater depth, particle radius, suspended solids, and dissolution rate were major drivers of risk estimates [150]. In contrast to conventional molecular ECs, whose fate is often approximated by partition coefficients and degradation rates, nano-Cu behavior is strongly governed by aggregation and sediment–water exchange, underscoring the need for nanospecific fate models.

Mechanistic studies further confirm that the hazard profile of engineered nanomaterials is dynamically shaped by water chemistry. Nano-Cu, nano-CuO, and Cu(OH)_2_ showed distinct aggregation, sedimentation, dissolution, and redox-transformation behavior across natural waters differing in ionic strength, pH, and organic content. Nano-Cu and Cu(OH)_2_ rapidly formed micrometer-scale aggregates, whereas nano-CuO remained comparatively more stable; however, aggregation did not directly predict sedimentation, indicating separate control by density, oxidation state, and surface chemistry. Dissolution increased under acidic conditions, while saline waters promoted insoluble chloride and carbonate phases. Even low phosphate concentrations altered surface charge and stabilized nano-CuO suspensions [151]. These findings demonstrate that nanomaterial mobility and hazard are not fixed intrinsic properties, but depend strongly on environmental geochemistry.

More broadly, a critical evaluation of engineered nanomaterial ecotoxicology emphasized that hazard testing is still often performed at concentrations exceeding realistic environmental levels, with insufficient consideration of aging, heteroaggregation, transformation, and realistic compartment partitioning. In addition, ENMs may act through both particulate and dissolved ionic forms, making dose metrics more complex than for conventional molecular ECs. Compared with PFAS or DBPs, where risk assessment is increasingly driven by concentration and structure, nanomaterial risk requires scenario-based evaluation that explicitly integrates exposure modeling, transformation dynamics, and body-burden considerations [152]. Overall, the environmental risk of molecular ECs is best understood through a limited number of cross-cutting mechanisms: chronic emission, incomplete removal, transformation-product formation, mixture effects, biological amplification, and matrix-dependent bioavailability. The analytical focus of this review is directly relevant to this risk interpretation because incomplete target lists, missing standards, ion suppression/enhancement, uncertain HRMS annotations, and poor recovery of particle-bound or highly polar compounds can all bias risk estimates. Consequently, future risk assessment should combine targeted quantification, suspect and non-target screening, transformation-product analysis, effect-based tools, and transparent uncertainty reporting. This approach provides a clearer synthesis than treating PFAS, pharmaceuticals, ARGs, nanomaterials, DBPs, rubber additives, and plastic-associated chemicals as isolated case studies.

### 6.2. Human Exposure Pathways and Health-Relevant Evidence

Human exposure to emerging contaminants is closely connected to water systems because contaminated surface water, groundwater, wastewater reuse, irrigation, aquaculture, and drinking-water treatment can transfer pollutants into drinking water, seafood, crops, dietary supplements, and other exposure media. In addition to direct exposure to parent compounds, transformation products, particulate-bound fractions, biofilm-associated contaminants, and mixtures can modify both exposure and toxicity. Therefore, the human health relevance of the analytical methods discussed in this review lies in their ability to connect water contamination with downstream exposure pathways, including drinking water, food webs, reuse systems, and biomonitoring evidence. Consequently, human health risk assessment increasingly requires integrated evaluation of chemical, biological, and environmental processes that influence contaminant mobility, bioavailability, transformation, and cumulative exposure.

The following examples are therefore not intended as separate exposure scenarios, but as connected outcomes of water-mediated contaminant transport and analytical detectability across environmental and human matrices. Human health risks from emerging contaminants increasingly arise through indirect and combined exposure pathways rather than from single contaminants in isolation. Micro- and nanoplastics are one example, because they can transport pathogens, antibiotic-resistant bacteria, resistance genes, and sorbed chemicals through biofilm formation and food-web transfer, thereby linking microbial and chemical exposure routes relevant to drinking water and seafood safety [34]. Chemical transfer through aquatic food webs is already evident for PFAS: in Portuguese commercial fish and bivalves, HRMS screening followed by targeted analysis showed that several samples exceeded EU maximum levels for PFOA under Regulation (EU) 2023/915, demonstrating that environmental contamination can translate directly into dietary exposure [97]. More broadly, recent syntheses indicate that soil health strongly influences whether biosolids- or wastewater-amended soils behave as sinks or secondary sources of micropollutants to groundwater and surface waters, reinforcing the need for integrated soil–water risk assessment in reuse systems [51].

MPs may also amplify toxin exposure by acting as concentration vectors for cyanotoxins. Adsorption studies showed up to 28-fold enrichment of microcystins on polymer surfaces, with maximum levels reaching 156 µg g^−1^ for the more hydrophobic congener MC-LF on polystyrene. Sorption was congener-dependent (MC-LF > MC-LR), polymer-specific (PS > PE > PVC > PET), and stronger for smaller particles, indicating dominant control by hydrophobic partitioning [35]. Because adsorption remained significant across pH 5–9, including bloom-impacted waters, these findings suggest that risk assessment based only on dissolved MC-LR may underestimate exposure, especially when particulate-bound fractions facilitate trophic transfer or create local hotspots.

This concern extends to water treatment, where contaminant removal does not necessarily equate to detoxification. For cyanotoxins, ozonation, chlorination, UV-based oxidation, and permanganate treatment show distinct kinetics, but structural degradation may still generate reactive intermediates, including halogenated products or aldehydic fragments from cleavage of toxic moieties such as the Adda group in microcystin-LR [11]. A related issue arises from nitrosamine precursor formation in biologically active waters. Controlled incubation experiments showed that microbial degradation of nitrogenous substrates increased NDMA formation potential by 2.6–7.9-fold before subsequent biodegradation reduced it again, with low-molecular-weight extracellular fractions (<1 kDa) dominating the precursor pool [153]. Because commonly monitored amines accounted for only a minor fraction, these results indicate that routine monitoring may miss biologically generated precursor reservoirs that become toxicologically relevant only after chloramination. Together, these findings show that process-dependent transformation can shift, rather than eliminate, human health risk during drinking water treatment.

Low-level pollution may also indirectly elevate human exposure by stimulating harmful cyanobacterial blooms. A recent synthesis reported a mean 57.9% increase in microcystin production under sub-toxic contaminant exposure, with most stimulatory responses occurring at ≤100 µg L^−1^ and many at ≤0.6 µg L^−1^. Antibiotics accounted for most documented cases, and stimulation was linked to the up-regulation of toxin biosynthesis and transport genes [154]. Thus, contaminants present below conventional toxicological thresholds may still enhance toxin burdens in source waters, increasing indirect human exposure through drinking water supplies.

Direct human exposure to cyanotoxins is not limited to drinking water. Commercial algal dietary supplements have been reported to contain microcystins up to 60 µg g^−1^, corresponding to estimated daily intakes up to 75-fold above the tolerable daily intake of 0.04 µg kg^−1^ body weight [111]. More broadly, chronic low-dose exposure may represent a more pervasive risk than episodic exceedances. Although WHO guidance values are based mainly on microcystin-LR, environmental matrices frequently contain mixtures of microcystins, cylindrospermopsin, anatoxin-a, and nodularin, as well as modified congeners whose toxic equivalency remains poorly defined [57]. Because routine monitoring still focuses on a limited subset of toxins, total cyanotoxin burden and mixture toxicity may be systematically underestimated across drinking water, food webs, and irrigation-related exposure pathways. This illustrates why HRMS-based congener profiling and effect-based assays are needed alongside targeted monitoring when cyanotoxin exposure extends from source waters to food and supplement products.

A similar structural undercoverage affects disinfection by-products. Large HRMS compilations list thousands of DBP structures, but only a minority have been confirmed with standards, indicating that exposure assessment remains biased toward analytically accessible compounds [4]. This gap becomes critical when highly toxic but previously unrecognized DBPs are formed. Recent GC–HRMS studies identified six alicyclic HCPDs in chlorinated and chloraminated drinking water, five of them newly reported as DBPs; one compound, 1,2,3,4,5,5-hexachloro-1,3-cyclopentadiene, showed in vivo toxicity values orders of magnitude higher than regulated THMs and HAAs and predicted bioconcentration factors of 384–3980 [19]. Even for regulated DBPs, population-scale risks remain measurable: a global THM synthesis reported a weighted mean concentration of 26.74 µg L^−1^ and an average lifetime cancer risk of 6.45 × 10^−5^, with a quantifiable contribution to bladder-cancer burden and marked regional disparities [155]. Thus, both regulated and unresolved DBP fractions remain relevant for long-term health risk in drinking water systems.

Emerging evidence also links non-classical contaminants to endocrine-related developmental outcomes. In a Chinese birth cohort, each doubling of early-pregnancy urinary 2-hydroxy-benzothiazole was associated with lower childhood BMI at age two, while tolyl-triazole and 2-hydroxy-benzothiazole were associated with reduced maternal free thyroxine and increased neonatal TSH. Mediation analysis further supported thyroid disruption as a mechanistic pathway [156]. These findings extend the human health risk picture beyond conventional carcinogenic or acute-toxic endpoints and indicate that low-level chronic exposure to traffic- and consumer-product-derived contaminants may also affect endocrine and developmental health.

Overall, these findings demonstrate that human health risks associated with emerging contaminants arise from connected water-mediated pathways, including drinking-water treatment, wastewater reuse, soil–water transfer, aquatic food webs, seafood consumption, dietary supplements, and internal exposure measured by biomonitoring. The analytical challenge is that each pathway may emphasize a different fraction of the contaminant burden: dissolved parent compounds in drinking water, transformation products after treatment, particle-bound contaminants in MPs, bioaccumulative residues in seafood, and metabolite biomarkers in human samples. This complexity highlights the limitations of monitoring strategies focused only on a restricted number of regulated analytes and supports integrated analytical and risk-assessment frameworks that combine targeted quantification, HRMS-based suspect/non-target screening, transformation-product analysis, particulate-fraction assessment, and biomonitoring across drinking water and food-exposure pathways.

### 6.3. Microplastics as Carriers and Modifiers of Contaminant Risk

Microplastic risk extends beyond simple particle occurrence alone and increasingly involves interactions with biological barriers, ecosystem processes, and co-occurring contaminants. Accordingly, risk assessment should address not only physical particle effects, but also the potential carrier role of MPs, indirect ecological disruption, and transfer between aquatic and terrestrial compartments.

From an analytical perspective, these risks are difficult to compare across studies because reported exposure depends strongly on particle size cut-off, polymer identification method, mass- versus number-based metrics, and contamination-control procedures. A recent One Health review further emphasized that micro- and nanoplastics should be considered persistent low-dose stressors across the lifespan, as growing evidence links their exposure to oxidative stress, inflammation, mitochondrial dysfunction, and cellular senescence, with potentially greater consequences in aging populations because of cumulative exposure and reduced physiological resilience [157].

Recent evidence has broadened concern beyond gastrointestinal and pulmonary exposure to include potential neurotoxicity of textile-derived micro- and nanoplastics. Fibrous particles released from synthetic textiles such as polyester, acrylic, nylon, and polyethylene have been detected in indoor air, household dust, and even human tissues, including brain samples [158]. Mechanistic studies suggest that nanoscale particles may reach the brain either through olfactory transport after inhalation or via systemic circulation and passage across the blood–brain barrier. Once internalized, they have been linked to oxidative stress, neuroinflammation, microglial activation, disruption of tight-junction proteins, and protein aggregation pathways associated with neurodegenerative disease [158]. These findings suggest that textile-derived MNPs should be considered not only as environmental particulates, but also as possible neurotoxic stressors, although exposure levels, particle characterization, and dose–response relationships still require further clarification.

At the ecosystem scale, MNPs also act as active modifiers of aquatic functioning rather than inert particles. Integrative analyses show that they interfere with carbon, nitrogen, sulfur, and phosphorus cycling by altering microbial community structure, enzyme activity, and redox microenvironments [34]. Such disturbances may impair nutrient sequestration, promote eutrophication, and reduce ecosystem resilience. In this sense, microplastic risk resembles that of other emerging contaminants, in that indirect ecological effects may be as important as direct toxicity.

Agricultural soils represent another important exposure compartment because of fragmentation of plastic mulching films. In a field study across Finland, Germany, and Spain, both conventional polyethylene mulch and biodegradable starch-blended PBAT mulch reduced microbial activities linked to soil functioning, particularly nitrogen cycling, with stronger effects after the second season, at higher loading (0.05% *w*/*w*), and in southern regions [159]. These results indicate that microplastic risks in soil depend on polymer type, exposure intensity, duration, and regional conditions, supporting the need for site-specific assessment.

MNPs also create biologically active plastisphere communities enriched in pathogens and antibiotic resistance genes. These biofilms can enhance microbial persistence and horizontal gene transfer, raising concern that microplastics may facilitate long-distance transport of microbial hazards and indirect exposure through drinking water and seafood pathways [34]. Compared with dissolved contaminants, microplastics therefore combine particulate, chemical, and microbiological risk dimensions within the same carrier.

Wastewater treatment may further shift these risks from water to land rather than removing them. A probabilistic risk assessment for sludge-amended soils derived an HC5 of 145 MPs kg^−1^ dw and suggested that microplastic contamination could affect 15–18% of soil species under realistic scenarios, compared with about 8% in control soils; under worst-case conditions, the potentially affected fraction rose to ~39% [32]. This indicates that apparent removal in WWTPs often represents redistribution to biosolids, reinforcing the need for regulatory thresholds for sludge intended for land application.

Overall, risks associated with microplastics arise not only from particle presence, but also from their capacity to cross biological barriers, disrupt ecosystem processes, host microbial hazards, interact with co-contaminants, and redistribute contamination between environmental compartments. These findings support integrated risk-assessment frameworks that combine particle number, size, shape, polymer type, mass-based metrics, biofilm-related hazards, adsorption behavior, and land–water transfer processes rather than relying solely on occurrence data.

## 7. Knowledge Gaps, Analytical Challenges, and Future Perspectives

Despite major progress in the analysis of ECs and MPs, several cross-cutting gaps still limit the translation of monitoring data into comparable and risk-relevant evidence. Rather than repeating the method-specific limitations discussed in previous sections, this section summarizes the main future priorities according to four integrated needs: representative monitoring, harmonized analytical workflows, improved interpretation of transformation and redistribution processes, and stronger linkage between occurrence, exposure, and risk.

First, monitoring strategies should better capture temporal and spatial variability. For molecular ECs, event-driven pulses, seasonal changes, precursor transformation, and reuse-related recirculation can strongly influence measured concentrations. Therefore, future monitoring should combine event-triggered, composite, passive, and multi-season sampling approaches according to source type and exposure scenario [17,79,94]. For MPs and tire-wear particles, harmonized sampling should include clear size cut-offs, contamination-control procedures, and, where possible, dual reporting of particle number and polymer or particle mass [91]. 

Second, analytical workflows require stronger harmonization across matrices and contaminant classes. For molecular ECs, targeted LC–MS/MS remains essential for reliable quantification, but it should be combined more systematically with HRMS-based suspect and non-target screening to capture transformation products, replacement PFAS, novel DBPs, tire-derived quinones, and other under-monitored compounds [23,24,25,26,27,28]. Sample preparation also remains a cross-cutting bottleneck because SPE, DLLME, MSPD, large-volume enrichment, and resin-based extraction are not equally suitable for all matrices and contaminant classes; future studies should therefore report matrix-specific recoveries, procedural blanks, isotope-labelled standard correction, matrix effects, and extraction losses more transparently [19,96,97,98,108]. For MPs and nanoplastics, particle-resolved spectroscopic methods and thermal-mass-based methods should be used as complementary tools, with transparent reporting of minimum detectable size, recovery, blank correction, polymer confirmation criteria, and QA/QC procedures [29,30,31,137].

Third, future studies should distinguish removal from redistribution or transformation. Apparent removal from water may reflect transfer to sludge, sediments, soils, biota, or concentrated residuals rather than true elimination. Similarly, degradation of parent compounds may generate transformation products with equal or greater persistence or toxicity. Monitoring frameworks should therefore consider parent compounds, transformation products, particulate-bound fractions, residual toxicity, and mass-balance indicators, especially in wastewater treatment, drinking-water treatment, and water-reuse systems [19,20,21,32,37,47].

Finally, risk interpretation remains constrained by incomplete structural identification, limited toxicological thresholds, mixture effects, uncertain bioavailability, and insufficient long-term exposure data. Future risk-relevant monitoring should integrate targeted quantification, HRMS-based identification confidence, MP particle and mass metrics, bioassays, exposure modeling, and ecological or human health indicators such as risk quotients, toxicity thresholds, and biomonitoring evidence [27,29,30,37]. Overall, future progress requires matrix-aware, harmonized, and risk-oriented monitoring frameworks that connect analytical chemistry with fate, exposure, toxicology, and regulatory decision-making for ECs and MPs in natural and treated waters.

## 8. Conclusions

This review highlights that reliable assessment of ECs and MPs in natural and treated waters requires integrated analytical frameworks that connect molecular contaminant analysis, particle characterization, exposure pathways, and risk interpretation. A central contribution of the review is the distinction between molecular ECs, which require sensitive chemical species analysis, and MPs, which require particle-resolved and polymer-mass-based characterization.

For molecular ECs, targeted LC–MS/MS remains indispensable for robust quantification of predefined contaminants and regulatory monitoring, whereas HRMS-based suspect and non-target screening is essential for expanding chemical coverage, identifying transformation products, replacement compounds, and previously unrecognized contaminants. For MPs, spectroscopic methods provide particle number, size, morphology, and polymer identity, while thermal-mass-based methods provide complementary polymer-mass information. Therefore, these approaches should be considered complementary rather than interchangeable.

The review also shows that occurrence data alone are insufficient for risk assessment. Sampling design, matrix-adapted sample preparation, QA/QC, identification confidence, and harmonized reporting strongly influence data comparability and the reliability of ecological and human health interpretation. In addition, apparent removal from water does not necessarily indicate risk reduction, because contaminants may be transformed into persistent or toxic products or transferred to sludge, sediments, soils, and reuse-related pathways.

Overall, the main priorities for future monitoring are harmonized sampling and reporting across matrices, integration of targeted quantification with HRMS-enabled suspect/non-target screening, combined particle-count and polymer-mass approaches for MPs and tire-wear particles, and stronger coupling between analytical chemistry, toxicology, bioavailability, and exposure assessment. These advances are necessary to transform analytical data into comparable, risk-relevant evidence for managing ECs and MPs in natural waters, wastewaters, reclaimed waters, and drinking-water-related systems.

## Figures and Tables

**Table 1:** Classified overview of sample-preparation strategies for molecular ECs.

Strategy Category	Typical Matrices	Main Advantages	Main Limitations
SPE and large-volume SPE/resin extraction	Surface water, wastewater, drinking water, groundwater, PFAS-impacted waters	High enrichment; suitable for trace-level LC–MS/MS and HRMS; widely used for target and suspect workflows	Sorbent bias, matrix effects, blank contamination, and lower throughput for large-volume extraction
DLLME and other miniaturized liquid extraction methods	Cleaner waters, seawater, selected biotoxin or hydrophobic EC applications	Rapid, low solvent use, good preconcentration, compatible with greener workflows	Less robust in lipid-rich or highly complex matrices; recovery strongly matrix-dependent
MSPD and solvent extraction with cleanup	Biota, seafood, fish tissues, bivalves, landfill refuse, sludge, soils	Combines extraction and cleanup; useful for heterogeneous and solid matrices	Extraction efficiency depends on homogenization, solvent, dispersant, and matrix composition
Soxhlet/PLE and cleanup-based extraction	Sediments, soils, sludge, biota, hydrophobic PCPs and UV filters	Efficient for strongly sorbed and hydrophobic compounds	Time/solvent intensive; co-extraction of matrix components; requires cleanup
Matrix-adapted and class-specific workflows	Saline waters, high-organic-matter samples, PFAS matrices, nanoparticle-containing samples	Allows correction of matrix effects; supports PFAS-specific contamination control or particle-preserving analysis	More laborious; requires strict QA/QC, standards, blanks, and method-specific validation

DLLME, dispersive liquid–liquid microextraction; ECs, emerging contaminants; HRMS, high-resolution mass spectrometry; LC–MS/MS, liquid chromatography–tandem mass spectrometry; MSPD, matrix solid-phase dispersion; PCPs, personal care products; PFAS, per- and polyfluoroalkyl substances; PLE, pressurized liquid extraction; QA/QC, quality assurance/quality control; SPE, solid-phase extraction.

**Table 2:** Concise comparison of the main analytical approaches discussed in this review. Detailed method-specific examples and references are provided in Supplementary Table S1.

Analytical Approach	Main Applicability	Typical Output/Sensitivity	Main Strengths	Main Limitations
LC–MS/MS	Targeted quantification of polar and semi-polar molecular ECs, including pharmaceuticals, PFAS, pesticides, sweeteners, and cyanotoxins	Usually ng L^−1^ to sub-ng L^−1^ for predefined analytes	High sensitivity, selectivity, reproducibility, and regulatory compatibility	Restricted to predefined targets; limited coverage of unknowns, transformation products, and non-standard congeners
GC–MS/GC–MS/MS	Volatile, semi-volatile, and thermally stable ECs, including selected DBPs, PAHs, PCBs, plasticizers, and neutral PFAS precursors	Usually ng L^−1^ or lower after suitable extraction	Strong complement to LC methods for GC-amenable compounds; useful structural information	Limited to volatile/semi-volatile and thermally stable compounds; derivatization may be required; less suitable for highly polar or thermolabile analytes
LC–HRMS/GC–HRMS	Suspect and non-target screening of molecular ECs, transformation products, DBPs, PFAS, and complex mixtures	Accurate-mass full-scan data; quantitative performance depends on standards and workflow	Broad chemical-space coverage; retrospective data mining; transformation-product discovery	Higher cost and data complexity; annotation uncertainty; limited quantification without authentic standards
LC–IMS–HRMS	Structural discrimination of isomers and closely related compounds, especially PFAS and complex suspect-screening workflows	Accurate mass, MS/MS, retention time, and collision cross section (CCS) information	Adds an orthogonal identification parameter; improves confidence in suspect screening	Requires IMS instrumentation and CCS libraries; interplatform CCS harmonization remains incomplete
µ-FTIR imaging	Particle-resolved MP analysis in water, sludge, sediment, and biota	Particle number, size, shape, and polymer identity; typically suitable for particles down to the low-µm range depending on instrument settings	Non-destructive; good polymer identification; supports particle counting and morphology assessment	Limited for very small MPs/NPs; interference from organic matter and weathered or black particles
µ-Raman spectroscopy	Particle-resolved identification of smaller MPs and selected nanoplastics	Polymer identity with higher spatial resolution than µ-FTIR	Useful for smaller particles; complementary to µ-FTIR	Fluorescence interference; slower mapping; possible thermal damage; matrix effects
Pyrolysis–GC–MS	Mass-based quantification of MPs, nanoplastics, and tire-wear particles	Polymer-specific mass concentrations, often reported as µg L^−1^ or µg g^−1^ depending on matrix	Suitable for complex matrices and dark/weathered particles; provides polymer-mass information	Destructive; no particle number, size, or morphology; polymer-specific markers and calibration required
TED–GC–MS	Thermal-mass-based screening of MPs in complex environmental matrices	Polymer-mass output after thermal extraction/desorption	Useful complement to spectroscopic methods; suitable for rapid polymer-mass screening	Destructive; limited particle-level information; possible overlap of thermal degradation products

CCS, collision cross section; DBPs, disinfection by-products; ECs, emerging contaminants; GC–HRMS, gas chromatography–high-resolution mass spectrometry; GC–MS/MS, gas chromatography–tandem mass spectrometry; HRMS, high-resolution mass spectrometry; IMS, ion mobility spectrometry; LC–HRMS, liquid chromatography–high-resolution mass spectrometry; LC–IMS–HRMS, liquid chromatography–ion mobility spectrometry–high-resolution mass spectrometry; LC–MS/MS, liquid chromatography–tandem mass spectrometry; MPs, microplastics; NPs, nanoplastics; PFAS, per- and polyfluoroalkyl substances; TED–GC–MS, thermal extraction–desorption gas chromatography–mass spectrometry; µ-FTIR, micro-Fourier transform infrared spectroscopy; µ-Raman, micro-Raman spectroscopy.

**Table 3:** Analytical strategies for particulate emerging contaminants (microplastics) in water matrices.

Analytical Strategy	Operational Size Range	Polymer Identification	Quantification Capability	Additional Information Obtained	Main Strengths	Main Limitations	Reference
Agglomeration with alkylated Fe_3_O_4_ + membrane filtration + Pyr–GC–MS	Nanoplastics; validated down to 20 nm for PS	PS and PMMA via characteristic pyrolysis products	Detection limits 0.02–0.03 µg L^−1^ for PS and PMMA; environmental PS detected in 11/15 samples at <0.07–0.73 µg L^−1^	Polymer-specific mass concentrations in environmental waters	Very low detection limits; efficient nanoplastic enrichment; magnetic agglomeration reduces filtration resistance	Destructive; no particle-size, shape, or count information; validated for limited polymer scope	[135]
Pressurized liquid extraction (PLE) + thermochemolysis (TMAH) + Pyr–GC–MS	Mass-based MP fraction (<5 mm); no particle-size resolution; applicable to biodegradable polymers and nanoplastic-containing extracts	PLA, PCL, PBS, PBAT, and PHAs	Recoveries 74–116%; LOQs 0.02–0.05 mg g^−1^; MDLs 0.06–0.17 µg injection^−1^; calibration R ≥ 0.95	Polymer-specific mass quantification with internal-standard correction	Simultaneous identification and quantification; suitable for complex matrices; thermochemolysis improves specificity	Destructive; no particle-number or morphology information; requires extraction and derivatization steps	[136]
TMAH digestion + dichloromethane dissolution + Pyr–GC–MS for solid matrices	Small MPs/NPs < 150 µm; validated down to 50 nm for PS and PMMA	PS, PMMA, PE, PP, PVC, PET	Recoveries 79.6–91.4%; size-dependent recoveries 69–101%; LODs 2.3–29.2 µg g^−1^; linearity R^2^ ≥ 0.97	Polymer-specific mass concentrations in soil, sediment, and sludge; total MNP levels 4.6–51.4 µg g^−1^ in real samples	Efficient extraction of small particles embedded in solid matrices; limited size effect; suitable for NOM-rich samples	Destructive; possible PVC-related benzene interference; centrifugation may cause minor nanoparticle losses	[137]
Pyr-GC-QqQ-MS in dynamic MRM mode with internal-standard calibration	Mass-based MP fraction (<5 mm); operational water fraction typically >5 µm after filtration; also applicable to nanoplastic-containing fractions	Twelve polymers including PMMA, PP, PVC, PA, PC, PA66, PE, PET, ABS, SBR, PUR, and PS	Nanogram-level sensitivity (1–126 ng depending on polymer); example LODs: 1 ng for PA, PUR, and PS; 7 ng for PET; 10 ng for PC; 12 ng for PP; 60 ng for PE	Polymer concentrations reported in multiple matrices: 2–35 µg L^−1^ in surface waters, 6.9–185.9 µg g^−1^ dw in sediments, and 0.1–13.3 mg g^−1^ dw in biota	Very high sensitivity and selectivity; internal-standard calibration; suitable for multiple matrices; CaCO_3_-assisted pyrolysis improves signal intensity	Destructive; no particle-number, size, or morphology information; requires specialized pyrolyzer–triple quadrupole instrumentation	[138]
Stormwater workflow combining filtration, FTIR, and Pyr–GC–MS for MPs and TWPs	Operational fraction > 25 µm	FTIR: PET and PP; Pyr–GC–MS: poly(butadiene) rubber and styrene–butadiene rubber (SBR) for TWPs	Inlet concentrations 3.8–59 MPs L^−1^ and 2.5–58 TWPs L^−1^ (0.4–4 mg L^−1^ TWPs); outlet concentrations 1.8–32 MPs L^−1^ and 1.3–32 TWPs L^−1^ (0.2–1.1 mg L^−1^ TWPs); removal 35–88%	Fragments dominated the MP profile (>94%); ~92% of fragments were black rubbery TWPs confirmed by Pyr–GC–MS; supports treatment-performance assessment	Combines particle counting with chemical confirmation; explicitly captures dense TWPs often missed by optical methods alone; directly useful for stormwater treatment benchmarking	Misses smaller MPs and NPs because of size cut-off; Pyr–GC–MS is destructive and increases analytical cost/time; FTIR is limited for black rubber; mass estimates remain method-dependent	[91]

MPs, microplastics; NPs, nanoplastics; TWPs, tire-wear particles; Pyr–GC–MS, pyrolysis–gas chromatography/mass spectrometry; FTIR, Fourier-transform infrared spectroscopy; TMAH, tetramethylammonium hydroxide; PS, polystyrene; PMMA, poly(methyl methacrylate); PE, polyethylene; PP, polypropylene; PVC, poly(vinyl chloride); PET, polyethylene terephthalate; PLA, polylactic acid; PCL, polycaprolactone; PBS, poly(butylene succinate); PBAT, poly(butylene adipate-co-terephthalate); PHAs, polyhydroxyalkanoates; PA, polyamide; PA66, polyamide 66; PC, polycarbonate; ABS, acrylonitrile butadiene styrene; SBR, styrene–butadiene rubber; PUR, polyurethane; NOM, natural organic matter; MRM, multiple reaction monitoring; LOD, limit of detection; LOQ, limit of quantification; MDL, method detection limit; dw, dry weight.

**Table 4:** Summary of key risk indicators and their relevance for EC and MP risk assessment.

Risk Dimension	Main Indicators	Relevance for Risk Interpretation	Analytical/Monitoring Implication
Risk characterization	Measured environmental concentration (MEC), predicted no-effect concentration (PNEC), risk quotient (RQ), toxicity thresholds	Identifies compounds, sites, or mixtures that may exceed ecological concern levels	Requires reliable quantification, harmonized target lists, matrix-specific QA/QC, and transparent reporting of detection limits
Transformation products	Parent-compound removal, transformation-product formation, toxicity change, persistence	Parent-compound disappearance does not necessarily indicate detoxification; some products may be more persistent or toxic than the parent compound	Requires targeted analysis combined with HRMS-based suspect and non-target screening
Mixture effects	Cumulative RQ, additive toxicity, synergistic or antagonistic responses, effect-based endpoints	Low individual concentrations may still contribute to combined biological effects	Supports mixture-based prioritization, bioassays, and effect-directed analysis
Antibiotic resistance	Antibiotic residues, antibiotic resistance genes (ARGs), mobile genetic elements (MGEs), co-selection markers	Represents transmissible biological risk rather than conventional chemical toxicity	Requires integration of chemical monitoring with molecular biological markers
MP carrier effects	Sorption/desorption, polymer type, particle size, weathering, biofilm formation, particulate-bound fractions	MPs may transport contaminants, additives, pathogens, and ARGs, but vector effects are context-dependent	Requires combined chemical–particle characterization and cautious interpretation of MP-associated risk
Human exposure pathways	Drinking water, wastewater reuse, crops, seafood, dietary supplements, biomonitoring	Connects water contamination with dietary, inhalation, dermal, and internal exposure	Requires multi-matrix monitoring from source water to treated water, food matrices, and human samples
Analytical uncertainty	Matrix effects, ion suppression/enhancement, missing standards, incomplete target lists, HRMS confidence levels	Can bias prioritization and underestimate total risk	Requires transparent uncertainty reporting, internal standards, recovery correction, and complementary targeted/HRMS workflows

ARGs, antibiotic resistance genes; ECs, emerging contaminants; HRMS, high-resolution mass spectrometry; MEC, measured environmental concentration; MGEs, mobile genetic elements; MPs, microplastics; PNEC, predicted no-effect concentration; QA/QC, quality assurance/quality control; RQ, risk quotient.

## Data Availability

No new data were created or analyzed in this study.

## Supplementary Materials

The following supporting information can be downloaded at: https://www.mdpi.com/article/10.3390/jox16030093/s1, Table S1. Comparative analytical strategies for molecular emerging contaminants in water and related matrices: instrumentation, analytical performance, identification capability, strengths, and limitations.
