# Supplementary material for: Analysis and Risks of Emerging Contaminants and Microplastics in Natural and Treated Waters and Human Health: A Critical Review
Source: J Xenobiot. 2026 May 23;16(3):93. doi: 10.3390/jox16030093 (PMC13302567; doi:10.3390/jox16030093)
Supplement: Supplementary file 1 [file jox-16-00093-s001.zip › jox-4266607-supplementary.pdf]

Journal of *Xenobiotics*

MDPI

# Supplementary Materials: Analysis and Risks of Emerging Contaminants and Microplastics in Natural and Treated Waters and Human Health: A Critical Review

**Maryam Mallek and Damia Barcelo**

**Table S1.** Comparative analytical strategies for molecular emerging contaminants in water and related matrices: instrumentation, analytical performance, identification capability, strengths, and limitations.

| Analytical strategy | Typical Instrumentation | Target EC Classes | Sensitivity / Analytical Performance | Identification Capability | Main Strengths | Main Limitations | References |
|---|---|---|---|---|---|---|---|
| Target + suspect screening of PFAS in multi-matrix waters (industrial hotspot) | UHPLC–Q Exactive Orbitrap HRMS with SPE | 77 target PFAS + ~120 suspects (PFCAs C4–C14, PFSAs, fluorotelomer sulfonates, betaines, precursors) | Low ng $L^{-1}$ quantification for targets; Σ77PFAS up to ~700 ng $L^{-1}$ in impacted waters | Accurate mass (±10 ppm), HRMS/MS, retention time, homolog patterns, isomeric signatures | Combines regulatory target analysis with suspect expansion; captures short-chain dominance and industrial source fingerprints; directly relevant to drinking-water exposure | Suspect results remain semi-quantitative; dependent on database coverage and blank correction; requires high-resolution instrumentation | [82] |
| NTA using LC–IMS–HRMS or GC–IMS–HRMS with unified confidence-level guidance | LC–IMS–HRMS or GC–IMS–HRMS platforms | PFAS | Not reported | Accurate mass, MS/MS, retention time, and CCS integrated into confidence Levels 1–5 | Incorporates CCS as an orthogonal identifier; improves transparency and harmonization; reduces ambiguity in PFAS annotation | No universal tolerance criteria; CCS variability depends on IMS platform; requires instrument-specific calibration and reporting | [118] |
| NTS for diffuse-source fingerprinting in estuarine sediments | UPLC–cIMS–QTOF–HRMS | Broad EC mixture (678 annotations; pharmaceuticals, food additives, pesticides, PFAS) | Not reported | Accurate mass + MS/MS + IMS-supported annotation | Captures underreported contaminants; supports hotspot and diffuse-source identification in complex sediments | Semi-quantitative without standards; annotation remains library-dependent; limited direct quantification | [119] |
| Integrated targeted quantification + suspect screening | UPLC–IMS–QTOF–HRMS with CCS-calibrated | Legacy and emerging PFAS in human serum, | Calibration range 0.1–100 ng $mL^{-1}$; serum concentrations | Targets: Level 1 using retention time, accurate mass, | Combines quantitative robustness with | Higher data-processing burden; generally, less | [81] |

 https://doi.org/10.3390/jox16030093

| | | | | | | | |
|---|---|---|---|---|---|---|---|
| + non-target profiling using LC–IMS–HRMS | library and isotope-dilution calibration | including PFOA, PFOS, HFPO-DA, F-53B, 6:2 DiPAP, PFPis, Cl-PFNPA | typically in the ng mL$^{-1}$ range | CCS, and isotope dilution; suspects: CCS + retention time + accurate mass; NTA for novel PFAS | multidimensional confirmation; supports longitudinal exposure assessment and retrospective discovery of replacement PFAS | sensitive than QQQ for ultra-trace targets; dependent on CCS libraries and serum-specific validation | |
| LC–IMS–QTOF–MS for rapid PFAS characterization in AFFF formulations | LC–IMS–QTOF–MS | Emerging PFAS in AFFFs, including structural isomers | Not reported | Accurate mass, MS/MS, and CCS for isomer and homolog-series discrimination | Multidimensional separation improves characterization of complex PFAS mixtures; enhances isomer discrimination; provides CCS-supported structural evidence | Requires IMS instrumentation; dependent on CCS library availability; no standardized confidence framework applied in the study | [123] |
| FluoroMatch IM software-assisted NTS for PFAS | LC–IMS–HRMS datasets processed with FluoroMatch IM | PFAS mixtures in environmental matrices | Not reported | Automated annotation using CCS matching, formula prediction, homologous-series detection, and mass-defect filtering | Open-source and vendor-neutral; integrates orthogonal CCS evidence; reduces manual review burden; supports expandable community libraries | Dependent on CCS library quality and calibration; requires prior feature extraction; annotation outcome depends on user-defined tolerance settings | [125] |
| Target and suspect screening enhanced by ion-mobility separation (CCS-supported IMS–HRMS) | LC–IMS–HRMS (QTOF-based platforms) with CCS library | Organic micropollutants in environmental waters | Not reported | Accurate mass, MS/MS, retention time, and CCS integrated into identification confidence; supported by an online CCS library of 556 compounds | Public CCS database strengthens target and suspect screening; CCS provides an instrument-independent descriptor; improves isomer discrimination | Application remains limited by CCS library availability and calibration quality; CCS is still not routinely incorporated into all identification frameworks | [122] |
| Total PFAS quantification by | Quantitative ^$^{19}$F-NMR with | Total PFAS including | Instrument LOD 99.97 nM (≈50 | Total fluorinated | Matrix-independent | Less sensitive than LC–MS; | [124] |

| | | | | | | | |
|---|---|---|---|---|---|---|---|
| ^$^{19}$F-NMR spectroscopy | internal standard and lyophilization preconcentration | ultrashort-chain PFAS (e.g., TFA) with terminal $-CF_3$ groups | μg $L^{-1}$ PFOS equivalent) | burden measured via characteristic $-CF_3$ signal; limited compound-level resolution | and free of ionization bias; captures poorly retained ultrashort-chain PFAS; does not require analyte-specific standards; avoids SPE-related bias | limited structural specificity in complex mixtures; restricted to PFAS containing terminal $-CF_3$ groups; requires complementary LC–HRMS for compound-level identification | |
| Targeted multi-residue analysis of pharmaceuticals in landfill refuse and leachate | LC–MS/MS (triple quadrupole) following refuse pretreatment and solvent extraction | Antibiotics and non-antibiotic pharmaceuticals in landfill refuse and leachate; 55 targets, 42 detected | MQLs: 0.40–16.8 ng $g^{-1}$ in refuse and 3 ng $L^{-1}$–1.24 μg $L^{-1}$ in leachate | Retention time + MRM transitions with isotope-labelled internal standards | Enables simultaneous quantification of broad pharmaceutical panels in heterogeneous landfill matrices; suitable for refuse–leachate partitioning and mass-balance studies | Restricted to predefined targets; matrix heterogeneity and strong sorption, especially for antibiotics, can affect extraction efficiency | [99] |
| Multidimensional LC–MS/MS with large-volume direct injection and heart-cutting separation | Multidimensional LC–MS/MS (triple quadrupole) with RP + HILIC–IE separation and dynamic valve switching | 60 PFAS from ultrashort- to long-chain homologues, including TFA | LOQ ≤1 ng $L^{-1}$ for most PFAS in surface and seawater; TFA LOQ ≈500 ng $L^{-1}$ | Targeted MRM quantification with simultaneous polarity-space expansion | Avoids offline SPE; enables simultaneous analysis of 60 PFAS across fresh and saline waters; reduces preparation bias for ultrashort-chain PFAS | TFA remains affected by background contamination and higher LOQ; instrumental setup is complex | [23] |
| HRMS confirmation of toxicant in exposure waters using archived extracts | LC–HRMS | Rubber-derived transformation products, especially 6-PPD-quinone | Not reported; concentrations reported as semi-quantitative because of matrix effects and detector saturation | Accurate-mass confirmation and semi-quantitative estimation of 6-PPD-quinone | Links chemical evidence directly to observed acute biological effects | Semi-quantitative only; no optimized quantitative method; matrix effects and detector saturation reduce robustness | [144] |
| Storm-event SPE coupled to | LC–MS/MS (triple quadrupole) | Tire-wear antioxidants and | 6-PPDQ <MDL–160 ng $L^{-1}$; 6PPD ≤130 ng $L^{-1}$; 1,3- | MRM with retention-time | Captures storm-driven | Restricted to target compounds; | [67] |

| | | | | | | | |
|---|---|---|---|---|---|---|---|
| targeted LC–MS/MS | with SPE: Oasis HLB (200 mg) | transformation products, including 6-PPD, 6-PPDQ, related PPDs/PPDQs, and 1,3-DMBA | DMBA 30–4800 ng $L^{-1}$; event-scale 6-PPDQ loads 0.078–2.7 g | and ion-ratio confirmation | concentration peaks and event loads; supports linkage between contaminant pulses and runoff hydrographs | analyte instability may affect results; some early measurements remained semi-quantitative | |
| Targeted LC–MS/MS verification of 6-PPD/6-PPDQ exposure concentrations in chronic toxicity experiments | LC–MS/MS (triple quadrupole) for exposure-water monitoring | 6-PPD and 6-PPD-quinone in experimental exposure waters | MDL 0.01 μg $L^{-1}$ for both analytes | Quantitative MRM-based confirmation of dosing concentrations | Provides robust verification of μg $L^{-1}$ exposure concentrations in controlled chronic assays; supports linkage between measured chemistry and biological responses | Restricted to predefined targets; specific to experimental matrix and renewal design; requires standards and strict QA/QC | [109] |
| Targeted LC–MS/MS with contamination-resilient workflow (delay-column configuration) | LC–MS/MS (triple quadrupole, MRM) with delay column and SPE/ultrasonication pretreatment | Phthalate diesters in water and solid matrices | LODs down to 0.2 ng $L^{-1}$ in water; sub-ng $L^{-1}$ to low ng $g^{-1}$ depending on matrix | Level 1 confirmation with standards and MRM transitions | High sensitivity; multi-matrix applicability (water, soil, waste, leachate); rigorous contamination control (delay column, blank correction); good precision (RSD <5%) | Restricted to predefined analytes; requires stringent contamination management; not suitable for unknown transformation products | [108] |
| High-throughput targeted exposomics using 96-well SPE–LC–MS/MS | LC–MS/MS (triple quadrupole) with 96-well SPE | >230 multi-class exposure biomarkers in urine, plasma, and serum | LOD <0.1 ng $mL^{-1}$ for 59–80% of analytes; recoveries 60–130%; RSD <30% | Targeted MRM-based quantitative or semi-quantitative analysis | Broad panel coverage with high throughput; suitable for large-cohort exposomics studies | Restricted to predefined analytes; matrix effects remain heterogeneous across chemical classes and matrices | [110] |
| Targeted LC–MS/MS following SPE preconcentration with standard | UPLC–ESI(−)–MS/MS (triple quadrupole) | Artificial sweeteners (acesulfame, saccharin, cyclamate, sucralose) | LOD: 0.006 mg $L^{-1}$; LOQ: 0.01 mg $L^{-1}$; $R^2$ > 0.995; RSD < 9.2% | Monitoring of $[M-H]^-$ ions (m/z 162, 178, 182, 395); compound-specific | Robust quantification in saline and coastal waters; good linearity and | Restricted to predefined targets; lower sensitivity than ng $L^{-1}$ PPCP | [53] |

| | | | | | | | |
|---|---|---|---|---|---|---|---|
| addition for saline matrices | | | | retention time confirmation | recoveries (73.5–128.4%); suitable for highly polar, persistent ECs | methods; matrix-dependent ionization requires standard addition | |
| Large-scale targeted monitoring of neonicotinoid pesticides in surface waters using direct aqueous injection | Direct aqueous injection LC–MS/MS (triple quadrupole) | Neonicotinoid pesticide (imidacloprid) in river water | Environmental concentrations: mean 24.9 ng $L^{-1}$, median 11.9 ng $L^{-1}$; 39% of samples exceeded the chronic benchmark of 10 ng $L^{-1}$ | MRM-based target confirmation with routine QA/QC | Enables continental-scale monitoring and long-term trend analysis across large sample sets | Restricted to predefined analytes; analytical performance may vary with matrix composition | [94] |
| Targeted quantitative monitoring of quaternary ammonium compounds in influent, effluent, and biosolids | UPLC–ESI(+)-QQQ-MS (triple quadrupole) with SPE | QAC homologues, including BACs, ATMACs, DADMACs, and EtBACs | Influent LOD 0.019–49 ng $L^{-1}$, LOQ 0.052–128 ng $L^{-1}$; effluent LOD 0.004–13 ng $L^{-1}$, LOQ 0.01–74 ng $L^{-1}$; biosolids LOD 0.019–49 μg $kg^{-1}$, LOQ 0.052–128 μg $kg^{-1}$ | Level 1 confirmation using authentic standards, dual MRM transitions, retention time, and ion-ratio verification | High sensitivity and selectivity; suitable for long-term multi-matrix surveillance; strong QA/QC and matrix-specific performance reporting | Restricted to predefined QAC homologues; requires recovery correction; not suitable for discovery of unknowns | [112] |
| Multiplex lateral flow assay for simultaneous marine-toxin screening | Gold nanoparticle–antibody conjugates on lateral-flow strip with portable reader | Marine biotoxins (OA, STX, DA) | LODs 0.1 ng $mL^{-1}$ (OA), 1.1 ng $mL^{-1}$ (STX), and 4.4 ng $mL^{-1}$ (DA) | Class-specific immunochemical recognition; semi-quantitative competitive assay | Rapid, portable, low-cost, and suitable for simultaneous multi-analyte screening | No structural confirmation; dependent on antibody specificity; lower analytical resolution than LC–MS/MS | [113] |
| Integrated bioassay–immunoassay–targeted and non-target LC–MS workflow for total microcystin characterization | LC–MS/MS + LC–HRMS + ELISA + PP2A inhibition assay | Microcystins and related cyanotoxins, including major and minor congeners | LC–MS/MS LOD 0.005 μg $g^{-1}$; reporting limit 0.01 μg $g^{-1}$; recovery >80% | Targeted MRM quantification for known congeners; HRMS for minor or reactive variants; bioassays for total PP2A inhibitory activity | Combines congener-specific quantification with broader toxicity screening; improves recognition of uncommon congeners; reduces underestimation from target-only analysis | Bioassays lack structural specificity; targeted LC–MS/MS may miss non-standard congeners; HRMS is less practical for routine monitoring; rare congeners may require surrogate calibration | [111] |
| Integrated target/suspect HRMS screening | Target PFAS: LC–MS/MS (triple | Legacy and emerging PFAS in soils, | Target MDLs from blank-based SD; | Target PFAS: Level 1 by MRM and | Combines robust target quantificatio | Workflow is complex and data-intensive; | [80] |

| | | | | | | | |
|---|---|---|---|---|---|---|---|
| with TOP assay validation | quadrupole); non-target: UPLC–Orbitrap HRMS; TOP assay | including ultrashort-chain PFAS and novel $CF_3$-containing compounds | instrumental LOQ = 10×S/N; Σ41PFAS 7.43–367 ng $g^{-1}$ dw; HFIP up to 854 ng $g^{-1}$ dw | standards; emerging PFAS: HRMS accurate mass, MS/MS, homologous-series/Kendrick filtering, and standard confirmation where available; precursor validation by TOP assay (TFA yields 8.87–40.0%) | n with structural discovery; resolves ultrashort-chain dominance; links precursors to transformation products; improves fluorine mass-balance interpretation | TOP assay reflects oxidative potential rather than actual environmental transformation; matrix-specific validation is needed beyond soils | |
| Integrated multistrategy suspect and non-target screening | UHPLC–QTOF–HRMS | PFAS and other fluorinated compounds in surface waters and WWTP effluents | Target PFAS quantified at ng $L^{-1}$ levels (surface water 2.3–45.3 ng $L^{-1}$; WWTP 6.1–65.6 ng $L^{-1}$); non-target features semi-quantitative | Level 1 for 36 PFAS with standards; Levels 2–4 for suspects based on MS/MS library matching, homologous-series consistency, spectral similarity, and reaction-guided annotation | Broad expansion of fluorinated chemical space beyond conventional PFAS lists; hierarchical evidence layering reduces false positives; molecular networking supports structural propagation | Data processing is intensive; strongly dependent on library completeness; non-target results remain semi-quantitative; full confirmation is limited by standard availability | [25] |
| Combined targeted and suspect screening using DLLME–LC–HRMS | UHPLC–QTOF–HRMS with DLLME preconcentration | Lipophilic marine biotoxins and 93 suspect derivatives | Seawater DLs 0.0004–1.7 ng $mL^{-1}$; mussel DLs 0.06–119 ng $g^{-1}$ | Accurate mass (±5 ppm), isotopic pattern, HRMS/MS fragments, retention time, and database matching | Combines target quantification with suspect screening; high sensitivity; suitable for marine matrices; supports detection of toxin derivatives during bloom events | Restricted by database coverage; no IMS/CCS support; structural isomers remain difficult to resolve; requires expert HRMS data processing | [96] |
| Combined targeted kinetics and HRMS-based transformation-product analysis during advanced oxidation | UHPLC–QqQ–MS for kinetics + UHPLC–QTOF–HRMS for TP identification | Acesulfame and chlorinated transformation products formed during UV/monochlo | Kinetic monitoring performed at µM level; TP detection enhanced at 25 µM parent concentration | Accurate mass and isotopic-pattern-based TP annotation | Enables identification of oxidant-specific transformation pathways beyond predefined | Requires elevated parent concentrations for TP detection; not directly suited to routine | [115] |

| | | | | | | | |
|---|---|---|---|---|---|---|---|
| | | ramine treatment | | | target lists; complements quantitative kinetic monitoring with structural TP discovery | environmental monitoring or compliance analysis | |
| Multiclass suspect screening using complementary LC–HRMS and GC–HRMS | LC–HRMS + GC–HRMS with large spectral libraries | Multiclass organic micropollutants, including pharmaceuticals, pesticides, personal care products, plasticizers, bisphenols, parabens, and industrial chemicals | LODs <1 to 100 ng mL$^{-1}$; 89% of QA/QC compounds ≤5 ng mL$^{-1}$ | 547 compounds annotated at Schymanski Levels 1–3; 63 confirmed at Level 1 with standards | Broad chemical-space coverage through LC/GC complementarity; high overall QA/QC detectability; applicable across environmental, food, and human matrices | Strong dependence on spectral-library completeness; no IMS/CCS support; confirmation limited by reference-standard availability | [126] |
| Multimatrix biota suspect screening using MSPD + paired LC/GC–HRMS, followed by targeted PFAS quantification | MSPD extraction with LC–QTOF–HRMS + GC–QTOF–HRMS; targeted UHPLC–MS/MS for PFAS | Multiclass organic pollutants in marine biota, plus PFAS of food relevance | No global NTS LOD/LOQ reported; targeted PFAS from LOQ to 5.8 ng g$^{-1}$ ww | 176 tentative organic pollutants, 77 confirmed with standards; LC– and GC–HRMS used complementary identification criteria; targeted PFAS quantified by LC–MS/MS | Strong chemical-space coverage in lipid-rich biota; MSPD is suitable for complex tissues; links suspect screening with food-regulatory PFAS assessment | NTS remains semi-quantitative; dependent on libraries and standard availability; targeted PFAS applied only to PFAS-positive subset | [97] |
| Suspect screening of pharmaceuticals and predicted transformation products in WWTP influent/effluent | UPLC–HRMS (Orbitrap) with full-scan and DDA MS/MS | Pharmaceuticals and predicted transformation products, including NSAIDs, antihistamines, antivirals, antitussives, and antibiotics | MQLs 0.18–154.5 ng L$^{-1}$; calibration range 0.01–500 ng mL$^{-1}$ | Accurate mass (<5 ppm), RT alignment (±0.2 min), and MS/MS library matching with confidence Levels 1–3 | Integrates suspect screening with TP prediction and RT prediction; enabled detection of 114 pharmaceuticals/TPs, including 13 newly reported WWTP TPs | Many TPs remain semi-quantified using parent compounds; large predicted candidate lists require substantial manual verification | [20] |
| Total fluorine screening + targeted PFAS | PIGE for total fluorine + LC–MS/MS for ionic | Ionic PFAAs, precursors, and neutral FTOHs in | Screening threshold >110 ppm total F; targeted | Total fluorine prioritization followed by compound- | Rapid identification of intentionally | Total fluorine lacks molecular speciation; | [104] |

| | | | | | | | |
|---|---|---|---|---|---|---|---|
| confirmation in consumer textiles | PFAS + GC–MS for neutral PFAS | reusable feminine hygiene products | Σ42PFAS 21–880 ng $g^{-1}$ in non-intentionally fluorinated products and 48–2205 ng $g^{-1}$ in intentionally fluorinated products | specific confirmation | fluorinated materials; captures both ionic and neutral PFAS classes | targeted analysis does not fully close fluorine mass balance | |
| Nationwide suspect and non-target screening with multi-extraction workflows and advanced data processing | UPLC–Orbitrap HRMS (ESI±) with DDA; multi-extraction cleanup and library/databank-supported annotation | Multiclass contaminants in sewage sludge, including pharmaceuticals, pesticides, personal care products, surfactants, PFAS, plasticizers, industrial chemicals, and natural products | No global LOD/LOQ for NTA features; Level 1 compounds calibrated at 0.1–1000 µg $L^{-1}$; sludge concentrations ranged from 0.2 ng $g^{-1}$ to 10,881 ng $g^{-1}$ dw | >500 contaminants identified; 382 confirmed at Level 1; additional Level 2 annotations by MS/MS library matching; homologous-series detection via Kendrick mass defect analysis | Large-scale nationwide coverage; broad chemical-space expansion through multi-extraction and homologous-series analysis; supports quantitative mass-load and per-capita flux estimation | Only a minority of features acquired MS/MS; strong matrix effects; low library-match rate; many features remained tentatively annotated; non-target results for non-standard compounds remain semi-quantitative | [28] |
| Integrated bioassay-guided suspect and non-target screening (virtual effect-directed analysis) in drinking water | LC–HRMS + GC–HRMS + ERα-CALUX bioassay + ICP–MS | Multiclass organic contaminants and trace elements in drinking water | Target MDLs based on S/N >3 or blank + 3×SD; MQLs based on S/N >10 or blank + 10×SD; maximum observed bioanalytical equivalent concentration 0.16 ng EEQ $L^{-1}$ | 16,929 aligned features after QA filtering; prioritized features ranked by statistical association with bioassay response; confidence assigned using Levels 1–5 | Integrates HRMS profiling with effect-based prioritization; complementary LC and GC coverage; transparent statistical workflow for feature prioritization | Most prioritized features remained tentatively annotated; correlation-based prioritization does not prove causality; no CCS dimension; non-target features remain semi-quantitative unless standards are available | [133] |
| GC–HRMS non-target analysis for alicyclic halocyclopentadienes formed during disinfection | GC–HRT–TOF–MS with complementary GC×GC–TOF–MS after large-volume resin enrichment | Volatile and semi-volatile halogenated DBPs formed during chlorination and chloramination | Discovery-based workflow; ng $L^{-1}$ occurrence levels reported; no routine LOD/LOQ established | Accurate mass, Cl/Br isotopic-pattern recognition, high-resolution EI fragmentation, library matching, and standard confirmation | High resolving power supports empirical-formula assignment; effective for halogen-rich GC-amenable DBPs; expands | Semi-quantitative in non-target mode; confirmation depends on standards; limited to GC-amenable compounds; structural isomers remain | [19] |

| | | | | | | | |
|---|---|---|---|---|---|---|---|
| | | | | for selected compounds | DBP space linked to unresolved TOX | difficult to resolve without standards | |
| Targeted quantification + HRMS elucidation of chlorination DBPs from bisphenols | UHPLC–QTOF–HRMS with complementary targeted quantification | Phenolic EDCs (bisphenol analogues) and reactive chlorination DBPs (BDA/BDAs) | Not primarily reported as LOD/LOQ-driven; time-resolved formation monitored over 120 min | Accurate-mass TP screening in dual polarity with targeted adduct quantification | Directly links parent contaminants to disinfectant-specific DBP formation; resolves pH dependence and chloramination effects | Requires derivatization for BDA detection; pathway-focused rather than broad screening workflow | [116] |
| Fragmentation-pattern-based non-target HRMS screening using diagnostic fragment filtering and neutral-loss-triggered $ddMS^2$ | UPLC–Orbitrap HRMS with complementary LC–MS/MS confirmation | Tire-derived PPD quinones, including known and newly identified PPD-Qs | Environmental concentrations reported at 0.02–0.21 μg $g^{-1}$ in tire tissue, 0.40–2.76 pg $m^{-3}$ in $PM_{2.5}$, and 0.23–1.02 ng $g^{-1}$ in surface soils | Accurate mass, diagnostic fragment ions, characteristic neutral losses, and MS/MS confirmation | Reduces false positives relative to suspect-only screening; supports discovery without predefined suspect lists; improves confidence for transformation products lacking standards | Requires high-resolution instrumentation and advanced processing; newly identified compounds remain semi-quantitative until standards are available; class-specific workflow | [26] |
| Combined target LC–MS/MS and LC–HRMS non-target screening with feature-based molecular networking for longitudinal river monitoring | Target: LC–MS/MS (triple quadrupole); non-target: UHPLC–Orbitrap HRMS | Pharmaceuticals, polymer-related chemicals, pesticides, consumer and industrial chemicals, and broader dissolved anthropogenic features | Target compounds quantified by external calibration and LOQ-based reporting; non-target features reported semi-quantitatively by peak-area scaling | Schymanski Levels 1–3 with library-based annotation and structural propagation through molecular networking | Integrates target and non-target evidence; supports large-scale feature coverage, robust blank filtering, and interpretation of longitudinal mixture dynamics and catchment influences | Non-target data remain semi-quantitative; many features remain unannotated; identification depends on library completeness; no absolute sensitivity metrics for NTS features | [127] |
| Target + suspect screening of polyhalogenated carbazoles | LC–HRMS with accurate mass and MS/MS fragmentation | Polyhalogenated carbazoles (Σ11PHCZs + unknown-related pollutants) | Compound-specific MDLs reported; measured concentrations ranged from 0.06–165.6 ng $g^{-1}$ dw in soils and 1.2–4.5 ng $g^{-1}$ dw in sediments | Accurate-mass target confirmation and suspect annotation with toxicity-equivalency evaluation | Enables detection of novel halogen substitution patterns; extends monitoring of dioxin-like | Restricted to one contaminant class; confirmation is limited without standards; interpretation remains | [128] |

| | | | | | | | |
|---|---|---|---|---|---|---|---|
| | | | | | contaminants to remote Arctic matrices; integrates toxic-equivalency interpretation | strongly HRMS-dependent | |
| Quantitative non-target screening of sewage sludge using nanoLC–HRMS with multi-database matching and isotopically assisted semi-quantification | NanoLC–Orbitrap HRMS with complementary ICP–MS | Organic micropollutants in biosolids, including pharmaceuticals, pesticides, flame retardants, industrial additives, natural compounds, and inorganic elements | No universal LOD/LOQ reported; feature detection threshold $10^6$ intensity; metal LODs 3×SD of blanks; semi-quantification median prediction error ~2.2–2.3-fold | 120 compounds identified, including 63 Level 1 and 57 Level 2 annotations; Schymanski framework with MS/MS similarity, accurate mass, and retention-time confirmation for Level 1 | High sensitivity in complex sludge extracts; rigorous QA/QC; broad database support; combines organic screening with semi-quantitative estimation and inorganic profiling | Semi-quantitative rather than fully quantitative; hydrophilic compounds are poorly retained on C18; PFAS not covered; dependent on library completeness; DDA may miss low-intensity precursors | [130] |
| Stable-isotope labeling with automated reactivity-directed non-target screening | HPLC–HRMS with H/D-glutathione probes and automated bioinformatics | Reactive disinfection byproducts in chlorinated and chloraminated waters | Conventional LOD/LOQ not reported; >50,000 raw features processed per dataset | 255 isotopically paired adducts screened; 202 DBPs annotated (193 newly reported); mechanistic classification (65 chlorinated substitution products; 132 unsaturated addition products) | Selectively prioritizes electrophilic and toxicologically relevant DBPs; supports automated high-throughput screening; enabled annotation of numerous previously unreported DBPs | Restricted to probe-reactive compounds; requires labeling chemistry; structural confirmation remains HRMS-dependent; not inherently quantitative | [131] |
| Entropy similarity-driven transformation reaction molecular networking (ESTRMN) | UPLC–Orbitrap HRMS for NTS with complementary LC–MS/MS for target quantification | Sartans and their wastewater transformation products | Target analytes quantified by validated LC–MS/MS; non-target features retained using entropy similarity >0.5 | 6 parent sartans confirmed at Level 1; 69 transformation products identified at confidence ≥ Level 3 using entropy similarity, diagnostic fragments, and knowledge- | Enables high-throughput transformation-product discovery; improves recovery of structurally related TPs; integrates transformation knowledge with confidence- | Limited to structurally related HRMS-detectable TPs; most TPs remain semi-quantitative; reliant on in-silico prediction and similarity thresholds; low-similarity products may be missed | [27] |

| | | | | | | | |
|---|---|---|---|---|---|---|---|
| | | | | guided prediction | based annotation and risk interpretation | | |
| Automated MS/MS data mining for non-target screening of ladder polyether toxins | UHPLC–QTOF HRMS/MS with complementary LC–MS/MS confirmation for selected toxins | Marine algal ladder polyether toxins from Gambierdiscus, including P-CTXs, gambierones, and related sulfated polyether-like features | Targeted gambierone LOD 0.09 ng $mL^{-1}$ and LOQ 0.3 ng $mL^{-1}$; non-target component is feature-based and semi-quantitative | MS/MS-pattern-based classification using sequential $H_2O/SO_3$ losses, diagnostic fragment ions, and accurate-mass matching to curated toxin datasets | Enables rapid extraction and classification of toxin-like features from large MS/MS datasets; supports family-level discovery when standards are limited; useful for strain-specific toxin fingerprinting | Dependent on data-acquisition quality; MS/MS alone cannot resolve structural isomers; confirmation of novel toxins still requires orthogonal evidence such as retention time, purification, or NMR | [134] |

AFFF, aqueous film-forming foam; ASCA, ANOVA-simultaneous component analysis; BACs, benzalkonium compounds; BDA/BDAs, brominated disinfection byproduct(s); cIMS, cyclic ion mobility spectrometry; CCS, collision cross section; $CF_3$, trifluoromethyl; Cl-PFNPA, chloroperfluorononylphosphonic acid; DADMACs, dialkyldimethylammonium compounds; DA, domoic acid; DDA, data-dependent acquisition; DBPs, disinfection byproducts; DL, detection limit; DMBA, dimethylbenzylamine; ECs, emerging contaminants; EEQ, estradiol equivalent; EDCs, endocrine-disrupting chemicals; EI, electron ionization; ER$\alpha$-CALUX, estrogen receptor alpha chemical-activated luciferase expression; ESI, electrospray ionization; EtBACs, ethylbenzalkonium compounds; ESTRMN, entropy similarity-driven transformation reaction molecular networking; FTOHs, fluorotelomer alcohols; GC, gas chromatography; GC×GC, two-dimensional gas chromatography; HILIC, hydrophilic interaction liquid chromatography; HRT-TOF-MS, high-resolution time-of-flight mass spectrometry; HRMS, high-resolution mass spectrometry; ICP-MS, inductively coupled plasma mass spectrometry; IE, ion exchange; IMS, ion mobility spectrometry; LC, liquid chromatography; LC–MS/MS, liquid chromatography–tandem mass spectrometry; $LC_{50}$, median lethal concentration; LOD, limit of detection; LOQ, limit of quantification; MDL, method detection limit; MDLs, method detection limits; MoNA, MassBank of North America; MQL, method quantification limit; MQLs, method quantification limits; MRM, multiple reaction monitoring; MS, mass spectrometry; MSPD, matrix solid-phase dispersion; nanoLC, nanoflow liquid chromatography; NMR, nuclear magnetic resonance; NSAIDs, non-steroidal anti-inflammatory drugs; NTA, non-target analysis; NTS, non-target screening; OA, okadaic acid; PFCAs, perfluoroalkyl carboxylic acids; PFAS, per- and polyfluoroalkyl substances; PFAAs, perfluoroalkyl acids; PFOS, perfluorooctane sulfonate; PFOA, perfluorooctanoic acid; PFSAs, perfluoroalkyl sulfonic acids; PFPis, polyfluorinated phosphinic acids; PFECAs, perfluoroether carboxylic acids; PIGE, particle-induced gamma emission; $PM_{2.5}$, particulate matter ≤2.5 μm; POPs, persistent organic pollutants; PP2A, protein phosphatase 2A; PPCP(s), pharmaceutical(s) and personal care product(s); QAC, quaternary ammonium compound; QACs, quaternary ammonium compounds; QQQ, triple quadrupole; QTOF, quadrupole time-of-flight; Ref., reference; RP, reversed phase; RSD, relative standard deviation; RT, retention time; Schymanski Levels, identification confidence levels for non-target screening; SD, standard deviation; SIM, selected ion monitoring; SPE, solid-phase extraction; STX, saxitoxin; TFA, trifluoroacetic acid; TOF, time-of-flight; TOP assay, total oxidizable precursor assay; TOX, total organic halogen; TP, transformation product; TPs, transformation products; UHPLC, ultra-high-performance liquid chromatography; UPLC, ultra-performance liquid chromatography; vEDA, virtual effect-directed analysis; WWTP, wastewater treatment plant.

https://doi.org/10.3390/jox16030093
